# Fabrication of Fructooligosaccharide‐*Larimichthys crocea* Peptide Copolymer by Wet‐Heating for Enhanced Calcium Delivery and Bone‐Lipid Metabolism Regulation

**DOI:** 10.1002/fsn3.71415

**Published:** 2026-01-02

**Authors:** Chunlei Liu, Xiaoping Wu, Yihang Guo, Dan Li

**Affiliations:** ^1^ College of Marine Science Ningde Normal University Ningde China; ^2^ Fujian Provincial University Engineering Research Center for Deep Processing of Mindong Aquatic Products Ningde China

**Keywords:** adipogenesis, calcium absorption, calcium delivery system, osteogenesis, physicochemical characteristics, wet‐heating

## Abstract

Calcium delivery systems based on food‐derived peptides and polysaccharides offer a promising strategy for improving calcium bioavailability while preserving peptide bioactivity. In this study, a fructooligosaccharide‐ *Larimichthys crocea* peptide (FOS‐LCP) copolymer was fabricated by wet‐heating and evaluated as a multifunctional calcium carrier. FOS‐LCP showed improved calcium‐binding capacity, digestive stability, and resistance to dietary inhibitors, thus increasing Ca^2+^ uptake and transport in intestinal models in vitro. Bone marrow mesenchymal stem cells isolated from calcium–deficient mice were used to assess bone‐lipid regulation in vitro. In the presence of Ca^2+^, FOS‐LCP promoted osteogenic differentiation and osteogenic marker expression, suppressed adipogenic differentiation, activated the Wnt/β‐catenin pathway, inhibited PPARγ/C/EBPα‐driven lipogenesis, reduced adipokine secretion, and enhanced lipolytic gene expression. These findings indicate that FOS‐LCP coordinately enhances calcium absorption and rebalances bone‐lipid metabolism at the cellular level, supporting wet‐heating‐derived peptide‐polysaccharide copolymers as promising functional calcium delivery systems.

## Graphical abstract

A food‐derived fructooligosaccharide‐ *Larimichthys crocea* peptide (FOS‐LCP) copolymer was prepared by wet‐heating and exhibited high calcium‐binding capacity and enhanced intestinal calcium uptake compared with native peptides. In bone marrow mesenchymal stem cells from calcium‐deficient mice, FOS‐LCP was found to promote osteogenesis while suppressing adipogenesis in vitro. These effects were associated with modulation of Wnt/β‐catenin signaling, supporting the potential of FOS‐LCP as a functional ingredient to improve calcium utilization and maintain bone‐lipid balance.

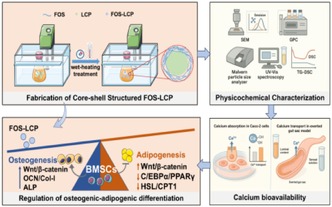

**Abbreviations**
ACC: acetyl‐CoA carboxylase
ALP: alkaline phosphatase
aP2: adipocyte fatty‐acid binding protein
ATGL: adipose triglyceride lipase
BGP: β‐glycerophosphate
BMSCs: bone marrow mesenchymal stem cells
C/EBPα: CCAAT/enhancer‐binding protein α
CaMKII: Ca^2+^/calmodulin‐dependent protein kinase II
Col–I: type I collagen
CPPs: casein phosphopeptides
CPT1: carnitine palmitoyltransferase 1
DEX: dexamethasone
DKK1: dickkopf‐1
DMT1: divalent metal transporter‐1
FAS: fatty acid synthase
FOS: fructooligosaccharides
GPC: gel permeation chromatography
GSK–3β: glycogen synthase kinase‐3β
HSL: hormone‐sensitive lipase
IBMX: 3‐isobutyl‐1‐methylxanthine
ICP–OES: inductively coupled plasma‐optical emission spectrometry
LCP:
*Larimichthys crocea* peptides
LRP5/6: low‐density lipoprotein receptor‐related protein 5/6
MMP7: matrix metalloproteinase–7
MW: molecular weight
OA: oxalate
OCN: osteocalcin
OPA: ortho‐phthalaldehyde
PA: phytate
PKC: protein kinase C
PPA: phosphate
PPARγ: peroxisome proliferator‐activated receptors γ
RT‐qPCR: reverse transcription quantitative polymerase chain reaction
Runx2: runt‐related transcription factor 2
SEM: scanning electron microscopy
SGF: simulated gastric fluid
SIF: simulated intestinal fluid
SPF: specific pathogen‐free
SREBP‐1c: sterol regulatory element‐binding protein‐1c
SSF: simulated salivary fluid
TG: triglyceride
TG‐DSC: thermogravimetry‐differential scanning calorimetry
UV‐Vis: ultraviolet‐visible spectroscopy

## 1 Introduction

Calcium is essential for skeletal homeostasis, with approximately 99% of body calcium stored in the bones, which slows bone mineral density loss and helps prevent bone diseases (Wu et al. 2022; Zhu et al. 2020). Nevertheless, approximately 51% of the global population is at risk of calcium deficiency owing to insufficient dietary intake and poor calcium bioavailability (Sun, Zhang, et al. 2020; Wu et al. 2022). Intestinal anti–nutritional factors, such as phytates (PA), oxalates (OA), and phosphates (PPA), readily form insoluble complexes with calcium, thereby reducing calcium absorption and contributing to bone‐related disorders (Wu, Wang, et al. 2023). Therefore, developing functional delivery systems that can overcome intestinal absorption barriers and enhance calcium bioavailability is a promising strategy for precise calcium supplementation and bone health maintenance.

Food‐derived calcium‐chelating peptides have attracted considerable interest because of their high calcium affinity and biocompatibility (Tian et al. 2025; Wu et al. 2024; Zhu et al. 2020). Among them, casein phosphopeptides (CPPs), the best‐studied representatives, chelate calcium via phosphoserine residues and facilitate intestinal uptake, but their phosphorylation‐dependent binding is weakened in the gastrointestinal tract because dephosphorylation and proteolysis destabilize peptide‐calcium complexes (Wu et al. 2024; Zhu et al. 2020). To enhance stability and delivery efficiency, peptide‐polysaccharide copolymers have been developed, in which polysaccharides serve as protective matrices around peptide‐calcium complexes (Wang et al. 2024; Wu, Wang, et al. 2023). Nevertheless, many reported systems employ neutral polysaccharides as inert scaffolds and rely on chemical coupling agents, which may limit their metabolic benefits and raise safety concerns regarding food applications (Mohamed et al. 2025). In this context, covalent peptide‐polysaccharide conjugation by wet‐heating offers a reagent‐free, food‐compatible route to improve copolymer stability under gastrointestinal pH and mechanical stress (Yang et al. 2024; Zhu et al. 2020).

Fructooligosaccharides (FOS) are attractive polysaccharide alternatives. As functional oligosaccharides, FOS exhibit physiological activities, including gut microbiota modulation, calcium absorption promotion, and lipid metabolism regulation (Ding et al. 2025; Kaewarsar et al. 2023; Zhang et al. 2021). Recent studies have shown that FOS act directly on the bone‐lipid axis. In high‐fat diet models, combined FOS/galactooligosaccharide (GOS) supplementation alleviates obesity‐related bone loss and reverses the skewed bone marrow progenitor differentiation by enhancing osteogenesis, suppressing excessive adipogenesis, and normalizing osteoclast formation, in parallel with gut microbiota restoration, short‐chain fatty acid production, intestinal barrier integrity, and systemic inflammation (Zhang et al. 2021). Nystose, a representative FOS, attenuates ovariectomy‐induced bone loss by promoting osteogenic differentiation in bone marrow mesenchymal stem cells (BMSCs) via BMP and Wnt/β‐catenin signaling and downregulating adipogenic gene expression in bone marrow (Zhang et al. 2023). Additionally, short‐chain FOS reduce body lipid accumulation, visceral adiposity, and insulin resistance in diet‐induced obesity models and overweight subjects by remodeling the gut microbiota and improving metabolic profiles (Ding et al. 2025; Yoo et al. 2024; Yu et al. 2019). Collectively, these findings confirm that FOS exert bidirectional control over bone‐lipid metabolism, simultaneously promoting osteogenesis and constraining excess adiposity at both the skeletal and systemic levels, which supports the selection of FOS as a polysaccharide moiety in a bone‐lipid‐targeted calcium delivery system (Zhang et al. 2024). Studies have also demonstrated that calcium is a key regulator of bone‐lipid homeostasis, influencing the commitment of BMSCs toward osteogenic or adipogenic lineages (Feng et al. 2024; Ntambi and Takova 1996; Seok et al. 2020). However, most peptide‐based calcium delivery systems primarily aim to increase intestinal uptake and rarely address this metabolic crosstalk (Tian et al. 2025; Wu et al. 2024). Therefore, integrating FOS with calcium–chelating peptides offers an opportunity to couple enhanced calcium bioavailability with active modulation of bone‐lipid balance.

Accordingly, we developed a multifunctional calcium delivery system based on a fructooligosaccharide‐ *Larimichthys crocea* peptide (FOS‐LCP) copolymer prepared by wet‐heating. First, we confirmed its calcium‐binding capacity and characterized its physicochemical properties using spectroscopy, scanning electron microscopy (SEM), and gel permeation chromatography (GPC). Second, calcium absorption and transport were evaluated using Caco‐2 cells and everted gut sac assays. Finally, BMSCs isolated from calcium‐deficient mice were used to elucidate the regulatory mechanisms of the copolymer in bone‐lipid metabolism in vitro. These findings are expected to inform the design of calcium delivery systems that are efficient, stable, and functionally synergistic and to open new avenues for applying aquatic‐derived bioactive peptides and functional oligosaccharides in precision nutritional interventions.

## 2 Materials and Methods

### 2.1 Materials

*L. crocea* was supplied by Ningde Guangyou Aquatic Products Co. Ltd. (Ningde, China). Insulin, 3‐isobutyl‐1‐methylxanthine (IBMX), β‐glycerophosphate (BGP), and dexamethasone (DEX) were products of Sigma–Aldrich (St. Louis, MO, USA). Alkaline phosphatase (ALP), type I collagen (Col‐I), osteocalcin (OCN), triglyceride (TG), glycerol, visfatin, and adiponectin ELISA kits were purchased from CLOUD‐CLONE Co. (Wuhan, China). FOS (purity ≥ 95%, degree of polymerization: 3–6) was procured from Shanghai Yuanye Bio‐technology Co. Ltd. (Shanghai, China). All other reagents were commercially available.

### 2.2 Calcium Delivery System Fabrication

Processed *L. crocea* surimi (16 g) was dispersed in ultrapure water (400 mL) and enzymatically hydrolyzed with flavourzyme (50°C, pH 7.0) for 2 h at a substrate‐to‐enzyme ratio of 1:25 (*w*/*w*) to obtain *L. crocea* peptides (LCP). The optimal conditions for preparing FOS‐LCP copolymers via wet‐heating were investigated by varying the LCP‐to‐FOS ratios (0.4:1–1.2:1), reaction pH values (9.0–13.0), temperatures (60°C–100°C), and reaction durations (1–5 h). Ultimately, a copolymer with an LCP‐to‐FOS mass ratio of 0.6:1 was synthesized at pH 11.0, 90°C for 2 h. All conditions were tested in three independent experiments with triplicates. The solution was dialyzed using dialysis membranes (molecular weight cut‐off: 3 kDa) at room temperature for 24 h and then lyophilized to obtain the FOS‐LCP copolymer. The degree of grafting of FOS onto LCP was determined based on the decrease in free amino groups measured using the o‐phthaldialdehyde (OPA) method, as described by Pirestani et al. (2017), and was calculated to be 50.29%.

### 2.3 Characterization of Physical and Chemical Properties

#### 2.3.1 GPC

The molecular weight (MW) distributions of LCP and FOS‐LCP were investigated using GPC (Agilent 1260 Infinity II, Agilent Technologies, Waldbronn, Germany) equipped with a TSKgel G2000SWXL column (7.8 × 300 mm^2^, 5 μm; Tosoh Bioscience, Tokyo, Japan) and a guard column, as described by Huang et al. (2023) with minor modifications. The mobile phase consisted of 50 mM sodium phosphate buffer containing 150 mM NaCl (pH 7.0) filtered through a 0.22 μm membrane and degassed by ultrasonication for 30 min. The flow rate and column temperature were maintained at 0.5 mL/min and 30°C, respectively. LCP and FOS‐LCP (0.5–1.0 mg/mL) were dissolved in the mobile phase and filtered through a 0.22‐μm PVDF membrane (Merck Millipore, Darmstadt, Germany) to remove particulates, and 20‐μL aliquots were injected. The detection was performed using a diode array detector set at 214 nm in series with a refractive index detector. Calibration was performed using 0.18–85 kDa pullulan standards (Sigma–Aldrich, St. Louis, MO, USA). A linear calibration curve was constructed, and the peak MW (Mp), number‐average MW (Mn), weight‐average MW (Mw), *z*‐average MW (Mz), and viscosity‐average MW (Mv) were calculated using the Agilent ChemStation software. Each sample was analyzed in triplicate from a single preparation.

#### 2.3.2 Particle Size Distribution Analysis

LCP and FOS‐LCP solutions (100 μg/mL) were filtered through a 0.22‐μm membrane, transferred to 1 cm polystyrene cuvettes, and equilibrated at 25°C for 5 min prior to measurement. The particle size distribution was analyzed using a laser particle size analyzer (ZEN3600, Malvern Instruments Ltd., Malvern, UK). Measurements were performed in triplicate per sample from a single preparation, with each run comprising 10 sub‐measurements to ensure data stability.

#### 2.3.3 SEM

Lyophilized FOS‐LCP and LCP powders (20 mg each) were evenly spread on an aluminum plate, and their microstructures were observed using SEM (Helios G4 CX, FEI, Brno, Czech Republic) at an accelerating voltage of 5 kV and a working distance of 8–10 mm according to Lin et al. (2020). To ensure the representativeness of microstructural features, the images were acquired at 10,000× and 20,000×. For each sample, three nonoverlapping regions were randomly selected and imaged. All SEM analyses were conducted on powders from a single independent preparation, and three nonoverlapping region images per sample were regarded as triplicates.

#### 2.3.4 INFOGEST Digestion

To evaluate the stabilizing effect of the FOS modification on calcium during gastrointestinal digestion, oral‐gastric‐intestinal in vitro digestion was performed following a standardized INFOGEST 2.0 procedure (Brodkorb et al. 2019) with minor modifications. Two test systems were prepared with the same initial total calcium: (i) FOS‐LCP + 5 mM CaCl_2_ and (ii) LCP + 5 mM CaCl_2_.

At the initial time point (*T*_0_), each test system (5 mL) was mixed with an equal volume of enzyme–free simulated salivary fluid (SSF) containing 13.6 mM KCl, 0.6 mM KH_2_PO_4_, 2.5 mM K_2_HPO_4_, and 15 mM NaHCO_3_ (pH 6.8) to simulate the oral phase matrix. The mixtures were centrifuged at 5000× *g* for 10 min at 4°C, and the supernatants were collected to determine the initial soluble calcium concentration ([Ca]_0_) using inductively coupled plasma optical emission spectrometry (ICP‐OES; iCAP 7400, Thermo Scientific, Waltham, MA, USA).

The digestion process comprised three sequential phases. The digestive fluids had electrolyte compositions consistent with INFOGEST 2.0, with CaCl_2_ omitted to avoid exogenous Ca^2+^ interference. Enzyme/bile blanks were run, and background Ca^2+^ was subtracted from all sample measurements. In the oral phase, SSF containing 75 U/mL α‐amylase was added to the test system to reach a final volume of 10 mL and incubated at 37°C for 2 min. For the gastric phase, simulated gastric fluid (SGF) containing 6.9 mM KCl, 0.9 mM KH_2_PO_4_, 25 mM NaHCO_3_, and 47.2 mM NaCl (pH 3.0) with 2000 U/mL pepsin was added to reach 20 mL and incubated at 37°C for 120 min. In the intestinal phase, simulated intestinal fluid (SIF) containing 6.8 mM KCl, 0.8 mM KH_2_PO_4_, 85 mM NaHCO_3_, 38.4 mM NaCl, and 0.5 mM MgCl_2_ (pH 7.0) with 100 U/mL pancreatin and 10 mM bile salts was added to reach 40 mL and incubated at 37°C for 120 min.

Aliquots (1 mL) were collected at 0 (pre‐digestion); 2 (end of oral phase); 30, 60, 90, and 120 min (gastric phase); and 150, 180, 210, and 240 min (intestinal phase). All samples were immediately cooled in an ice bath for 10 min, then centrifuged at 5000× *g* for 10 min at 4°C. Supernatants exceeding the ICP‐OES linear range were diluted with ultrapure 1% HNO_3_ before analysis. Soluble calcium concentrations ([Ca]_*t*_) were determined by ICP‐OES. All experiments were performed in triplicate using samples from at least three independent preparations. Soluble calcium retention rate was calculated as follows:

$$\text{Soluble calcium retention}\;\left(\%\right)=\left(\frac{{\left[\mathrm{Ca}\right]}_{t}\times V_{t}}{{\left[\mathrm{Ca}\right]}_{0}\times V_{0}}\right)\times 100\%$$

where [Ca]_*t*_ is the soluble calcium concentration at time “*t*,” *V*_*t*_ is the total volume at time “*t*,” [Ca]_0_ is the initial soluble calcium concentration at *T*_0_, and *V*_0_ was 10 mL.

#### 2.3.5 Thermogravimetric Analysis‐Differential Scanning Calorimetry

The LCP and FOS‐LCP powders (5 mg each) were placed in a high‐temperature crucible. Under a 30 mL/min argon gas flow rate, the samples were heated from 50°C to 500°C at 10°C/min. The mass loss and heat change of samples during the entire heating process were recorded using thermogravimetry‐differential scanning calorimetry (TG–DSC; STA449C/6/G, NETZSCH, Selb, Germany) as described by Lin et al. (2020). All measurements were conducted in triplicate from a single preparation.

#### 2.3.6 Ultraviolet‐Visible Spectroscopy

LCP and FOS‐LCP (10 mg each) were dispersed in ultrapure water (100 mL, pH 7.0) and filtered (0.22 μm) prior to measurement. Ultraviolet‐visible (UV‐Vis) spectra were recorded at 25°C on a UV‐2600 spectrophotometer (Shimadzu, Kyoto, Japan) with the following parameters: scanning range 190–400 nm, bandwidth 1 nm, and data interval 1 nm (Mo et al. 2023). Ultrapure water served as a blank, and the spectra were baseline–corrected against the blank. All measurements were performed in triplicate from a single preparation.

#### 2.3.7 Fluorescence Spectroscopy

Lyophilized FOS‐LCP and LCP powders (10 mg each) were dispersed in ultrapure water (100 mL, pH 7.0) and filtered (0.22 μm) prior to measurement. Then, the fluorescence spectra were measured at 25°C using a fluorescence spectrometer (FluoroMax‐4C, Horiba Instruments Inc., Edison, NJ, USA) with the following parameters: excitation wavelength 295 nm, emission wavelength range 300–500 nm, excitation and emission slit widths of 5 nm. Ultrapure water served as a blank and the spectra were baseline‐corrected against the blank, as described by Han et al. (2024). All measurements were performed in triplicate from a single preparation.

### 2.4 Calcium Absorption Assay

#### 2.4.1 Cell Culture and Cytotoxicity Assay

Caco‐2 cells were cultured in DMEM supplemented with 10% (*v*/*v*) FBS, 1% (*v*/*v*) nonessential amino acids, and 1% (*v*/*v*) penicillin/streptomycin at 37°C in a humidified atmosphere containing 5% CO_2_. When cells reached 90% confluence, they were detached using 0.25% trypsin‐EDTA and seeded at a density of 5 × 10^3^ cells/well in 96‐well plates. After 24 h attachment, the medium was replaced with 100 μL fresh medium containing 0–25 mM CaCl_2_, 0–25 mg/mL LCP, or 0–25 mg/mL FOS‐LCP and incubated for 24 h. Fresh medium without samples served as the control. Cell viability was assessed using the MTT assay (Lin et al. 2020). Data represent three independent experiments, each performed in triplicate.

#### 2.4.2 Calcium Uptake Under Inhibitor Intervention

Dietary inhibitors, including OA, PA, PPA, FeSO_4_, and ZnSO_4_ were selected as factors that interfere with calcium absorption. Caco‐2 cells were detached using 0.25% trypsin‐EDTA and seeded in 12‐well plates at a density of 1 × 10^5^ cells/well. After incubation for 2 days, different concentrations of CaCl_2_, CaCl_2_/FeSO_4_, CaCl_2_/PA, CaCl_2_/PPA, CaCl_2_/OA, CaCl_2_/ZnSO_4_, LCP/CaCl_2_, LCP/CaCl_2_/FeSO_4_, LCP/CaCl_2_/PA, LCP/CaCl_2_/PPA, LCP/CaCl_2_/OA, LCP/CaCl_2_/ZnSO_4_, FOS‐LCP/CaCl_2_, FOS‐LCP/CaCl_2_/FeSO_4_, FOS‐LCP/CaCl_2_/PA, FOS‐LCP/CaCl_2_/OA, FOS‐LCP/CaCl_2_/PPA, and FOS‐LCP/CaCl_2_/ZnSO_4_ were used to treat the cells, where the concentration of CaCl_2_, FeSO_4_, or ZnSO_4_ was 5 mM; LCP or FOS‐LCP was 8 mg/mL; and the mass ratio of OA, PA, and PPA to CaCl_2_ was 20:1 (*w*/*w*). The medium–only group served as the control. After 2 h of incubation, the cellular calcium content was determined according to Lin et al. (2020). Briefly, cells were washed twice with HBSS, and Fluo‐3AM (5 μM) was loaded by adding 100 μL per well and incubating at 37°C for 1 h. Fresh HBSS was then added, and the cells were incubated for 30 min to allow de‐esterification to Fluo‐3. Fluorescence was analyzed using a flow cytometer (BD Accuri C6 Plus; BD Biosciences, San Jose, CA, USA) with excitation at 488 nm and emission at 525 nm. The intracellular calcium concentration ([Ca^2+^]*ᵢ*) was expressed as fluorescence intensity relative to the control. All conditions were tested in three independent experiments with triplicates.

#### 2.4.3 Calcium Transport

The mouse ileum was used as an experimental model for the everted gut sac to investigate calcium transport enhancement by LCP and FOS‐LCP. Twelve‐week‐old healthy female ICR mice (25 ± 1 g, *n* = 5) were purchased from Hangzhou Medical College (Hangzhou, China). All trials were conducted in accordance with the “Guidelines for the Care and Use of Experimental Animals” authorized by the Fujian Provincial Laboratory Animal Management Office and approved by the Ningde Normal University Animal Care and Use Committee (approval number: NDNU‐LL‐202507). After 1 week of adaptive feeding, the mice were fed a low‐calcium diet (1 g CaCO_3_/kg diet) for 8 weeks to induce calcium deficiency. During this period, they were housed in a specific pathogen‐free (SPF) environment and underwent a 12 h light/dark cycle at 24°C ± 1°C and 40%–60% relative humidity, with free access to deionized water. After 12 h of fasting, the mice were anesthetized with 1% (*m*/*v*) pentobarbital sodium (40 mg/kg body weight, intraperitoneal injection) before euthanasia, and bilateral femurs and ileum were collected. The body weights of the mice are listed in Table S1.

Calcium transport assays were performed according to Sun, Wang, et al. (2020) with minor modifications. Briefly, the ileum segment (approximately 7 cm, 2 cm proximal to the ileocecal junction) was flushed with ice–cold saline and transferred to oxygenated Krebs–Ringer bicarbonate buffer at 37°C with continuous gassing (95% O_2_/5% CO_2_). One end was then ligated, and the segment was gently everted on a glass rod. The lumen was filled with 1 mL calcium‐free Krebs–Ringer bicarbonate buffer containing 10 mM glucose and the other end was ligated. Sacs were immersed in 6 mL mucosal buffer containing 5 mM CaCl_2_ with or without 2–8 mg/mL LCP or FOS‐LCP and incubated at 37°C for 120 min under continuous gassing (95% O_2_/5% CO_2_). Mucosal buffer containing 5 mM CaCl_2_ served as a control. After incubation, the serosal fluid was collected and the transported calcium was quantified by ICP‐OES. Each condition used ≥ 5 sacs from ≥ 3 mice.

### 2.5 BMSC Isolation and Culture

BMSCs were isolated and cultured according to Zhang et al. (2021) with minor modifications. Briefly, the femurs and tibias were excised from the hind limbs of calcium–deficient mice and placed in 6‐well plates containing ice‐cold PBS after removing the surrounding muscles. Both ends of each bone were cut to open the marrow cavity, and bone marrow cells were flushed with culture medium using a syringe needle. DMEM supplemented with 10% (*v*/*v*) FBS and 1% penicillin/streptomycin was then added to the 6‐well plates, and the cells were cultured at 37°C in 5% CO_2_. After 24 h, the culture medium was replaced to remove nonadherent cells and was refreshed every 2 days until the cultures reached approximately 95% confluence, at which point the cells were passaged. Third‐passage BMSCs (P3) were used for subsequent experiments. At approximately 90% confluence, P3 cells were detached and seeded in 96‐well plates at a density of 5 × 10^3^ cells/well. After 24 h, they were incubated in medium containing 5 mM CaCl_2_ with or without 25–400 μg/mL FOS‐LCP for 24–72 h. A medium containing 5 mM CaCl_2_ served as the control. Cell viability was assessed using MTT assay (Lin et al. 2020). Each experiment was performed in triplicate using cells from at least three independent mice.

### 2.6 Osteogenic and Adipogenic Differentiation

Osteogenic differentiation was performed according to Wu et al. (2020) with minor modifications. Briefly, 1 × 10^5^ BMSCs/well were seeded in 6‐well plates and incubated at 37°C in 5% CO_2_ for attachment. When the cultures reached approximately 80% confluence, the medium was aspirated and replaced with osteogenic differentiation medium (DMEM containing 10% (*v*/*v*) FBS, 1% (*v*/*v*) penicillin/streptomycin, 5 mM BGP, 100 nM DEX, and 50 μM ascorbic acid) with 100–400 μg/mL FOS‐LCP and 5 mM CaCl_2_ at 37°C in 5% CO_2_ for 7, 14, or 21 days, with the medium refreshed every other day. The calcium‐only control group received osteogenic differentiation medium containing 5 mM CaCl_2_ without FOS‐LCP and was cultured on the same schedule.

Adipogenic differentiation was performed according to Wu, Chen, et al. (2023) with minor modifications. Briefly, 1 × 10^5^ BMSCs/well were seeded in 6‐well plates and incubated at 37°C in 5% CO_2_ for attachment. When the cultures reached 90% confluence, the medium was replaced with fresh medium containing 100–400 μg/mL FOS‐LCP and 5 mM CaCl_2_ and further incubated for 2 days. The medium was then switched to adipogenic differentiation medium (DMEM containing 10% (*v*/*v*) FBS, 10 μg/mL insulin, 5 μM troglitazone, 1 μM DEX, 0.5 mM IBMX, and 1% (*v*/*v*) penicillin/streptomycin) with 100–400 μg/mL FOS‐LCP and 5 mM CaCl_2_ for an additional 2 days. Then, the BMSCs were cultured in maintenance medium (DMEM containing 10% (*v*/*v*) FBS, 10 μg/mL insulin, and 1% (*v*/*v*) penicillin/streptomycin) with 100–400 μg/mL FOS‐LCP and 5 mM CaCl_2_ for 8 days, with maintenance medium refreshed every other day. A calcium‐only control was included throughout adipogenic induction and maintenance, in which cells received the corresponding medium containing 5 mM CaCl_2_ and were cultured in parallel. Each experiment was performed in triplicate using cells from at least three independent mice.

### 2.7 Alizarin Red S and Oil Red O Staining

Alizarin red S staining was performed to evaluate mineralized calcium nodules after 21 days of differentiation, according to Wu et al. (2022). Briefly, the cells were fixed with 4% (*w*/*v*) paraformaldehyde for 10 min at room temperature and stained with 40 mM alizarin red S solution (pH 4.2) for 30 min. The unbound dye was removed by rinsing with sterile water and images were acquired under a microscope. To quantify the mineralized nodules, the cells were destained with 10% (*w*/*v*) cetylpyridinium chloride in dark for 1 h, and the absorbance of the eluted dye was measured at 570 nm using a microplate reader.

Oil red O staining was performed to assess lipid accumulation in differentiated adipocytes, according to Wu et al. (2023). After differentiation, the cells were fixed with 4% (*w*/*v*) paraformaldehyde for 20 min, briefly pretreated with 60% isopropanol for 30 s, stained with filtered oil red O solution according to the manufacturer's instructions, and imaged under a microscope. The bound dye was eluted with isopropanol and the absorbance was measured at 490 nm using a microplate reader. The quantitative results were normalized to the total protein content determined using the BCA assay. Each experiment was performed in triplicate using cells from at least three independent mice.

### 2.8 ALP Activity Assay

BMSCs were induced to differentiate for 7 or 14 days with 100–400 μg/mL FOS‐LCP in the presence of 5 mM CaCl_2_. A calcium‐only control group was included, in which the cells were treated with 5 mM CaCl_2_ under the same conditions. Then, the cells were lysed in 1% Triton X‐100 by repeated freeze–thaw cycles (three times at −80°C/37°C), followed by ultrasonication (300 W, 5 s on/10 s off, 3 cycles) on ice and centrifugation at 12,000× *g* for 10 min at 4°C to collect the supernatant. Cellular ALP activity was detected using a commercial ALP assay kit according to the manufacturer's instructions. Each experiment was performed in triplicate using cells from at least three independent mice.

### 2.9 OCN, Col–I, and Adipokine Production

After the BMSCs were induced to differentiate for 7 or 14 days in the presence of 100–400 μg/mL FOS‐LCP mixed with 5 mM CaCl_2_, the supernatants were collected and centrifuged at 1000× *g* for 5 min at 4°C to remove the cellular debris. OCN and Col‐I production were measured using commercially available ELISA kits following the manufacturer's instructions. A calcium–only control group was included, in which cells were induced with 5 mM CaCl_2_ under the same conditions. Likewise, after differentiation with 100–400 μg/mL FOS‐LCP mixed with 5 mM CaCl_2_ for 10 days, the supernatants were harvested to measure adipokine (adiponectin and visfatin) concentrations using ELISA kits according to the manufacturer's instructions. Each experiment was performed in triplicate using cells from at least three independent mice.

### 2.10 Intracellular TG Content and Lipolysis Assay

After differentiation with 100–400 μg/mL FOS‐LCP mixed with 5 mM CaCl_2_ for 10 d, cells and culture media were collected. For TG measurement, the cells were lysed in 1% Triton X‐100 by repeated freeze‐thaw cycles (three times at −80°C/37°C), followed by ultrasonication (300 W, 5 s on/10 s off, 3 cycles) on ice and centrifugation at 12,000× *g* for 10 min at 4°C to collect the supernatant. The TG content in the cell lysate was determined using a commercial TG assay kit following the manufacturer's guidelines. The collected medium was incubated at 70°C for 10 min to inactivate the residual lipases (Wu, Wang, et al. 2023), and the glycerol content in the medium was determined according to the manufacturer's instructions. A calcium‐only control group was included in which cells were induced with 5 mM CaCl_2_ and TG and glycerol levels were measured in parallel under the same conditions. Each experiment was performed in triplicate using cells from at least three independent mice.

### 2.11 Reverse Transcription Quantitative Polymerase Chain Reaction

BMSCs were differentiated with 100–400 μg/mL FOS‐LCP mixed with 5 mM CaCl_2_ for 7 days to investigate osteogenesis‐related gene expression. For adipogenesis‐related gene expression, BMSCs were differentiated in the presence of 100–400 μg/mL FOS‐LCP with 5 mM CaCl_2_ for 10 days. A calcium‐only control group was included, in which the cells were differentiated with 5 mM CaCl_2_ under identical conditions. At the end of differentiation, total RNA was extracted using the TRIzol reagent (Invitrogen, Carlsbad, CA, USA) according to the manufacturer's instructions. RNA purity and concentration were assessed using a spectrophotometer, and 1 μg total RNA was reverse‐transcribed to cDNA using a commercial reverse transcription kit (Takara Bio Inc., Shiga, Japan). Reverse transcription quantitative polymerase chain reaction (RT‐qPCR) was performed using the SYBR Green Master Mix (Applied Biosystems, Foster City, CA, USA) on a QuantStudio 6 Flex Real‐Time PCR System (Applied Biosystems). 18S rRNA was used as an internal reference gene. The amplification conditions were as follows: initial denaturation at 95°C for 5 min, followed by 40 cycles of 95°C for 10 s and 60°C for 30 s. The relative target gene expression levels were calculated using the 2^−ΔΔCt^ method. Each sample was analyzed in triplicate and the experiments were repeated with cells from at least three independent mice. The primer sequences are listed in Table S2.

### 2.12 Statistical Analysis

All experiments were performed in triplicate with at least three biological replicates (*n* = 3), and results were expressed as mean ± standard deviation (SD). Statistical analyses were conducted using SPSS software (version 22.0; IBM Corp., Armonk, NY, USA). One‐way analysis of variance (ANOVA) was used to assess differences among groups, followed by Duncan's multiple range test for post–hoc comparisons. *p* < 0.05 was considered statistically significant.

## 3 Results and Discussion

### 3.1 Fabrication of Calcium Delivery System

Wet‐heating is an efficient conjugation strategy for fabricating calcium delivery systems, primarily because the incorporated polysaccharides protect peptides from protease degradation, whereas their hydroxyl groups provide additional calcium‐binding sites (Wu, Wang, et al. 2023; Zhu et al. 2020). As a prebiotic, FOS exhibits excellent stability and compatibility, thereby enhancing calcium absorption in vivo (Kaewarsar et al. 2023). Therefore, the effects of reaction conditions, such as the LCP/FOS mass ratio, reaction time, temperature, and pH, on the calcium‐binding capacity of the FOS‐LCP copolymer were investigated.

**FIGURE 1:**
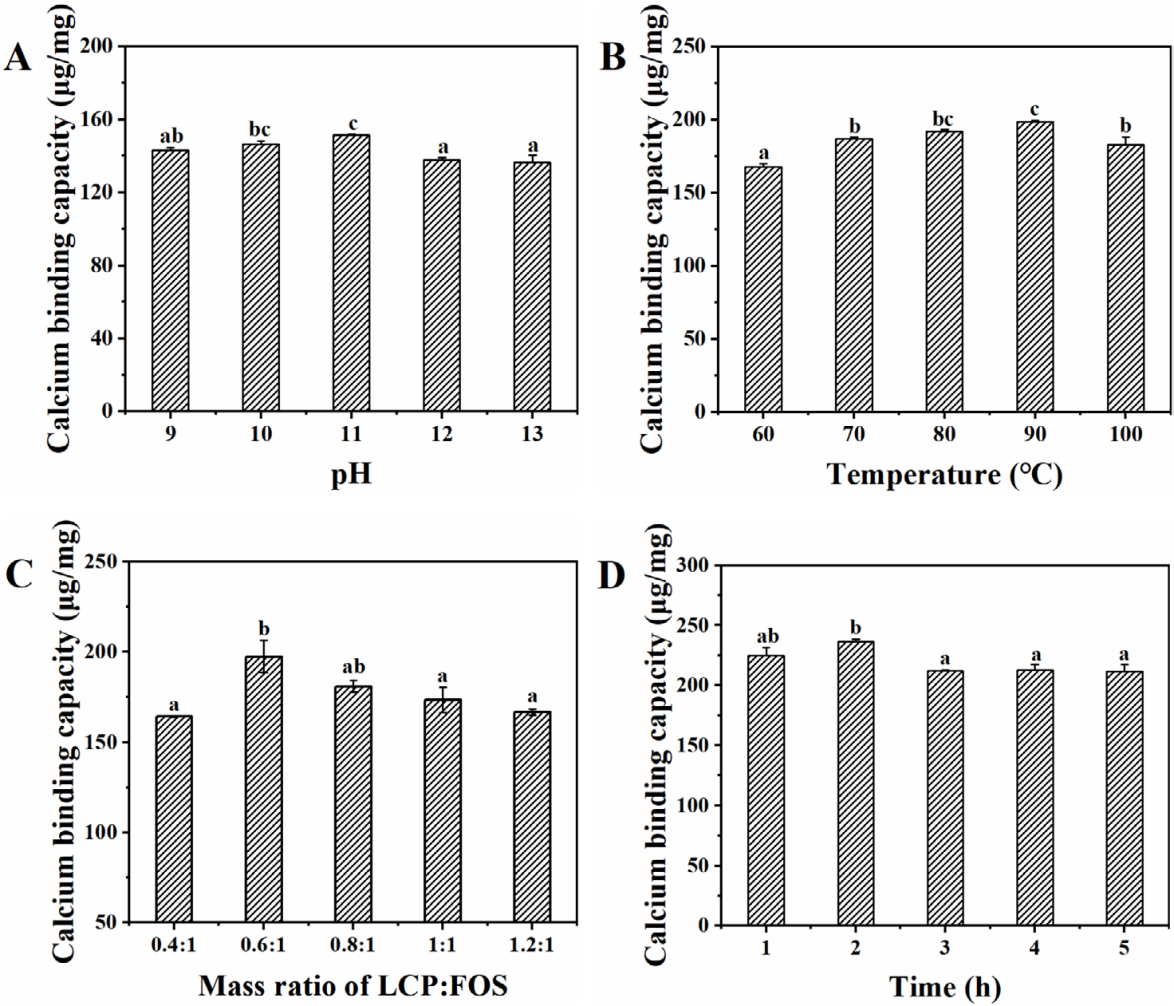
Effect of pH (A), temperature (B), *Larimichthys crocea* peptides (LCP): fructooligosaccharides (FOS) mass ratio (C), and time (D) on the calcium‐binding capacity of the FOS‐LCP copolymer under wet‐heating treatment. Each condition was tested using three independent preparations (biological replicates, *n* = 3), each with technical triplicates. Different lowercase letters indicate significant differences among treatment groups (*p* < 0.05).

The copolymer's calcium‐binding capacity reached a maximum of 151.54 μg/mg at pH 11.0 (Figure 1A), possibly because alkaline conditions favor the wet‐heating reaction (Zhu et al. 2020). However, when the pH was increased further, the calcium‐binding capacity decreased. This may be attributed to peptide cross–linking and aggregation under strongly alkaline conditions, which reduces the degree of grafting and limits the number of effective calcium‐binding sites (Jiang et al. 2021). Similarly, the calcium‐binding capacity peaked at 90°C (Figure 1B) because moderate heating promotes the Amadori rearrangement reaction (Pirestani et al. 2017). Nevertheless, when the temperature was elevated to 100°C, peptide folding and aggregation likely occurred, impairing the carbonylation reaction of ammonia required for copolymer formation and thus reducing calcium‐binding efficiency (Jiang et al. 2021). This trend is consistent with the grafting reaction of CPP‐dextran systems reported by Jiang et al. (2021). Different peptide‐to‐saccharide ratios affect system dispersion, and thus, the interactions between FOS and LCP (Wang et al. 2024). As the LCP concentration increased, the copolymer's calcium‐binding capacity followed a bell–shaped curve and reached a maximum (197.52 μg/mg) at a ratio of 0.6:1 (Figure 1C). At higher LCP levels, steric hindrance and reduced interactions led to FOS‐limited copolymer formation (Jiang et al. 2021). Reaction time further modulated the binding performance. Initially, when both FOS and LCP were present at relatively high concentrations, molecular interactions were favored, resulting in the highest calcium‐binding capacity (236.57 μg/mg) after 2 h (Figure 1D). However, a prolonged reaction led to excess glycation, which progressively decreased the available calcium‐binding sites and ultimately lowered the binding capacity of the copolymer (Jiang et al. 2021).

### 3.2 Physicochemical Characterization

#### 3.2.1 MW Distribution Analysis

**FIGURE 2:**
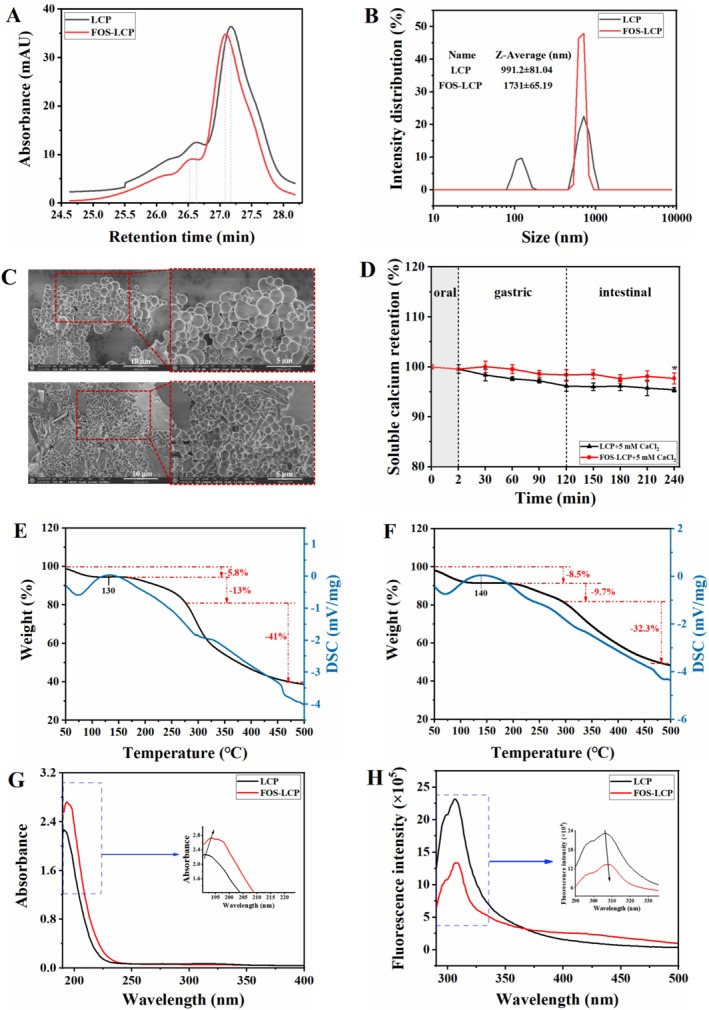
Structural and physicochemical characterization of the calcium delivery system. (A) Gel permeation chromatography (GPC) elution profiles; (B) particle size distribution; (C) scanning electron microscopy (SEM) images at 10,000× and 20,000× magnifications (top panel: LCP; bottom panel: FOS‐LCP); (D) in vitro digestive stability; (E) thermal stability of LCP determined by thermogravimetry‐differential scanning calorimetry (TG‐DSC); (F) thermal stability of FOS‐LCP determined by TG‐DSC; (G) ultraviolet‐visible (UV‐Vis) absorption spectra; (H) fluorescence spectra. Panels A, B, C, and E–H: technical triplicates from a single preparation. Panel D: three independent preparations (biological replicates, *n* = 3), each with technical triplicates; “*” indicates a significant difference between the FOS‐LCP group and the LCP group at the same time point (*p* < 0.05).

The elution profiles of LCP and FOS‐LCP determined by GPC are shown in Figure 2A. Both LCP and FOS‐LCP exhibited two elution peaks, and both peaks of FOS‐LCP shifted toward shorter elution times, a shift that was attributed to covalent conjugation, which generated larger molecular species during the wet‐heating treatment (Lin et al. 2020). The MW averages shown in Table 1 further support this observation. In particular, the Mn of peak 1 for LCP was 987 g/mol, indicating that LCP mainly consists of short oligopeptides (Lin et al. 2020). Previous studies have shown that peptides and saccharides with low MW are more likely to exhibit stronger calcium‐binding capacities owing to reduced steric hindrance, which facilitates the interaction between calcium ions and chelating sites (Lin et al. 2020). After grafting with FOS, the Mn increased to 2750 g/mol, but the copolymer remained within the low MW range, thereby retaining a favorable calcium‐binding capacity (Pirestani et al. 2017; Qi et al. 2023). In addition, the degree of grafting of FOS onto LCP, calculated from the OPA‐based determination of free amino groups, reached 50.29%, indicating that a substantial fraction of the amino groups in the peptide mixture participated in covalent conjugation with FOS under optimized wet‐heating conditions. Since LCP is an enzymatic hydrolysate containing a heterogeneous peptide mixture, this grafting degree reflects the overall extent of FOS conjugation at the mixture level rather than the residue–specific modification of a single defined peptide sequence; nonetheless, it provides chemical evidence supporting FOS‐LCP conjugate formation. However, the present characterization did not resolve the specific glycosylation sites or amino acid residues involved in FOS conjugation, and future work using LC‐MS/MS‐based peptide mapping will be required to delineate these residue–level modification patterns.

**TABLE 1:** Molecular weight averages of LCP and FOS‐LCP.

Sample	Peak	Mp (g/mol)	Mn (g/mol)	Mw (g/mol)	Mz (g/mol)	Mv (g/mol)
LCP	1	901	987	1058	1142	1129
	2	598	426	461	489	485
FOS‐LCP	1	1833	2750	3014	3328	3279
	2	576	697	811	937	919

*Note:* Mn refers to the number—average molecular weight; Mp refers to the peak molecular weight; Mv refers to the viscosity—average molecular weight; Mw refers to the weight—average molecular weight; Mz refers to the viscosity—average molecular weight.

#### 3.2.2 Particle Size Analysis and SEM Observation

The SEM images of LCP and FOS‐LCP revealed closely connected spherical particles that formed grape–like clusters with smooth surfaces (Figure 2C; the corresponding original high‐resolution images are provided in Figure S1). This morphology may be attributed to the self‐assembly ability of LCP and FOS‐LCP, which facilitates encapsulation of calcium ions and protects against environmental influences, thereby enhancing bioavailability (Zhao et al. 2020). Notably, the average particle size of LCP was 991.2 nm, whereas grafting with FOS increased it to 1731 nm (Figure 2B). This enlargement was attributed to Amadori bond formation, which induced the molecular folding and aggregation of FOS and LCP (Zhao et al. 2020; Zhu et al. 2020). Moreover, the particle size distribution of LCP exhibited a bimodal pattern, whereas the FOS‐LCP copolymer displayed a concentrated unimodal distribution, indicating that the copolymer particles were more uniform, a finding that was visually corroborated by the SEM images (Figure 2C). Taken together, these results suggest that LCP was effectively encapsulated and stabilized by FOS, whereas Amadori bond‐mediated polymerization further optimized the structural organization of the copolymer (Wu et al. 2022; Zhu et al. 2020).

#### 3.2.3 Digestive Stability

As potential calcium supplements, peptide‐polysaccharide delivery systems are intended for oral administration and, therefore, are exposed to diverse gastrointestinal factors, including digestive enzymes (Lin et al. 2020). Thus, the soluble calcium retention of LCP and FOS‐LCP during simulated digestion was evaluated (Figure 2D). The retention rates of both systems were > 95% throughout the digestion process, indicating their intrinsic stability in the gastrointestinal environment. Notably, FOS‐LCP displayed a modest but significant increase in soluble calcium retention compared with LCP at the intestinal endpoint (240 min, *p* < 0.05), which was accompanied by consistently higher retention across all intestinal stages (150–240 min). These findings suggest that FOS conjugation may provide a protective barrier to prevent calcium precipitation and maintain calcium in its soluble form (Gao et al. 2018). A similar stabilizing effect was reported by Zhu et al. (2020), in which desalted duck egg white peptides complexed with chitosan oligosaccharides helped sustain peptide–calcium systems under gastrointestinal conditions.

Although the absolute difference between the two systems is limited, the consistent trend of higher soluble calcium retention supports a modest but significant stabilizing effect of FOS conjugation on peptide–calcium complexes under digestive conditions (Wang et al. 2024). This represents indirect evidence based on soluble calcium retention and therefore does not directly demonstrate peptide integrity, which is a limitation of the present study. SDS–PAGE or LC‐MS/MS analyses before and after digestion will be required in future studies to confirm the protection against enzymatic degradation (Jiang et al. 2024). Nevertheless, even a modest increase in soluble calcium levels may be physiologically relevant. Previous research has demonstrated that incremental improvements in calcium retention can accumulate over time to support long‐term bone health, particularly in groups with marginal calcium intake, such as vegans and older adults with reduced calcium absorption (Tong et al. 2020; Torfadottir and Uusi‐Rasi 2023).

#### 3.2.4 TG‐DSC

Many compounds undergo changes in heat flow and mass during thermal decomposition, and a combination of these indicators can be used to analyze physicochemical changes (Lin et al. 2020). Therefore, TG‐DSC can be employed to analyze the thermal stability of LCP and FOS‐LCP. A pronounced endothermic peak was observed near 70°C on the DSC curves of both LCP and FOS‐LCP without corresponding mass loss in the TG curves (Figure 2E,F), likely reflecting the evaporation of adsorbed water (Pirestani et al. 2017). Moreover, a major endothermic peak occurred at 130°C for LCP and 140°C for FOS‐LCP, with the peak for FOS‐LCP exhibiting a greater weight loss. This difference may be attributed to the cleavage of additional C–N bonds introduced by glycosylation at elevated temperatures (Lin et al. 2020). Notably, the total weight loss for LCP and FOS‐LCP over the entire heating process was 59.8% and 50.5%, respectively, indicating that a larger proportion of chemical bonds were disrupted in LCP, leading to more extensive decomposition. These findings suggest that FOS can covalently bind to LCP during glycosylation and the resulting FOS‐LCP copolymer exhibits stronger intermolecular interactions and enhanced thermal stability than LCP. Together with the OPA‐based grafting analysis (grafting degree 50.29%) and GPC results, these thermal data further support the conclusion that FOS is covalently conjugated to LCP in the FOS‐LCP copolymer.

#### 3.2.5 UV‐Vis Spectroscopy Analysis

The combination of specific functional groups alters the energy requirements of electronic transitions, thereby affecting the peak position and intensity of the UV–Vis spectra (Wu, Wang, et al. 2023). After grafting FOS onto LCP, the maximum absorption peak shifted from 191 to 194 nm, with the peak intensity increasing from 2.25 to 2.76 (Figure 2G), indicating a bathochromic shift accompanied by increased absorbance. The red shift was probably caused by the conjugation between the lone pair electrons of –OH and –NH_2_ groups and *π* electrons of the C = O bond (Wu et al. 2022). In addition, the formation of Schiff bases may have altered the spatial structure of certain amino acid residues, such as aromatic amino acids, thereby exposing more chromophores and leading to increased UV absorbance (Jiang et al. 2018). These results indicate that the FOS‐LCP copolymer is a novel conjugate with structural characteristics that are distinct from those of LCP.

#### 3.2.6 Fluorescence Spectroscopy Analysis

Conformational changes in copolymers and their interactions with metal ions can be elucidated by changes in fluorescence intensity (Lin et al. 2020). After conjugation with FOS, the fluorescence peak intensity of LCP decreased markedly at approximately 310 nm (Figure 2H), possibly due to FOS‐LCP formation, which disrupted the conjugated double bonds of LCP and induced substituent effects (Wu, Wang, et al. 2023). Moreover, the appearance of fluorescence peaks at 400–420 nm in the FOS‐LCP copolymer further confirms successful conjugation between FOS and LCP (Wu et al. 2022).

### 3.3 Calcium Absorption–Promoting Activity

#### 3.3.1 Calcium Absorption Under Dietary Inhibitors

**FIGURE 3:**
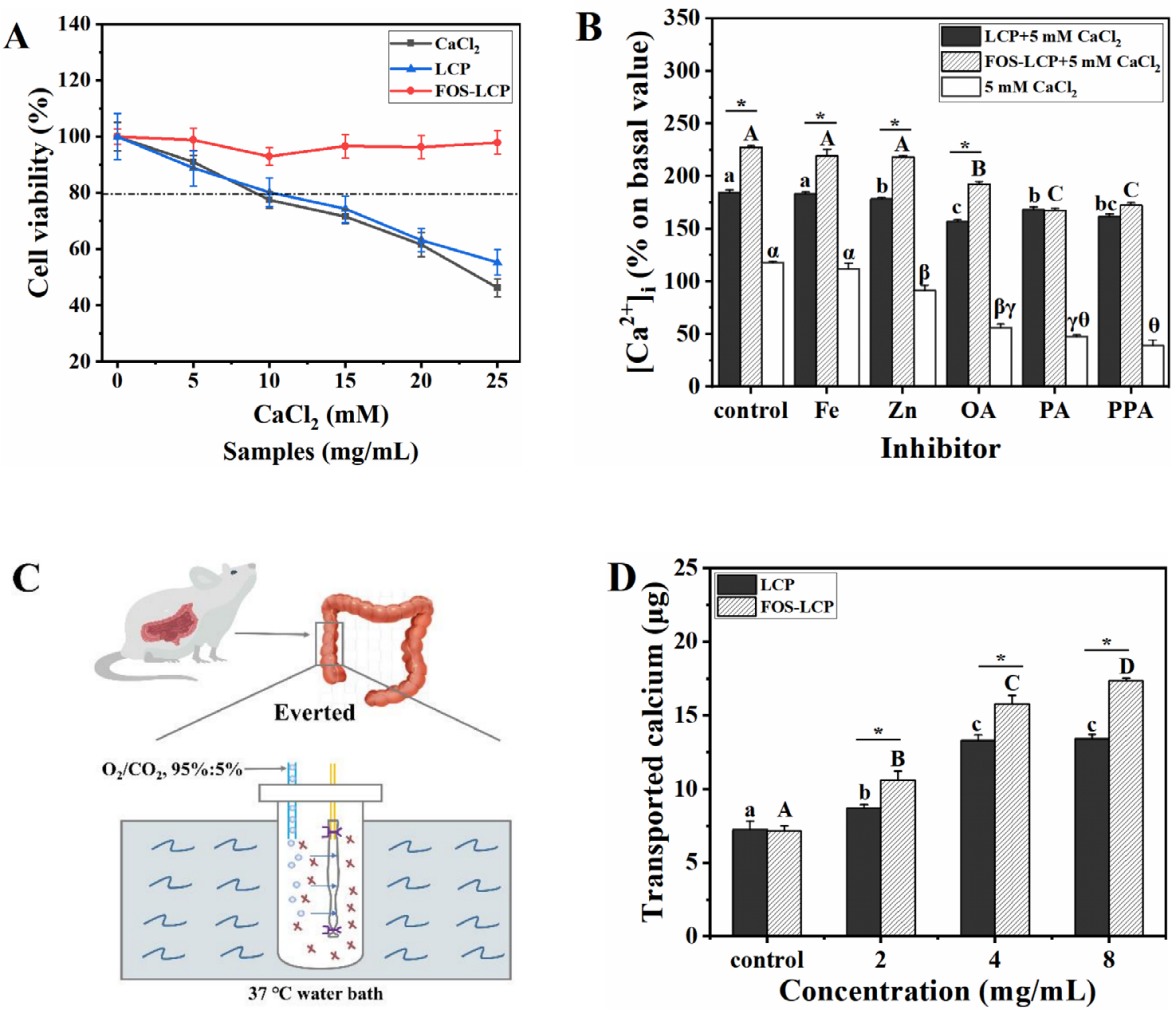
Calcium absorption‐promoting activity of FOS‐LCP. (A) Cell viability of Caco‐2 cells treated with different concentrations of CaCl_2_, LCP, or FOS‐LCP (control: medium only); (B) calcium absorption in the presence of dietary inhibitors (control: medium only); (C) schematic of the everted gut sac model; (D) transported calcium across everted gut sacs in the presence of different concentrations of LCP or FOS‐LCP (control: 5 mM CaCl_2_ only). Panels A, B, D: three independent biological replicates (*n* = 3) with technical triplicates. Different uppercase letters indicate significant differences among concentrations within the FOS‐LCP group (*p* < 0.05). Different lowercase letters indicate significant differences among concentrations within the LCP group (*p* < 0.05). “*” denotes a significant difference between LCP and FOS‐LCP at the same treatment (*p* < 0.05).

Caco‐2 cells were used to evaluate the effects of LCP and FOS‐LCP on cellular calcium uptake in the presence of an additional calcium source. Prior to the absorption assays, the cytotoxicity of CaCl_2_, LCP, and FOS‐LCP was assessed using the MTT assay. The viability of cells treated with 0–7.5 mM CaCl_2_, 0–10 mg/mL LCP, or 0–25 mg/mL FOS‐LCP was above 80% (Figure 3A). Therefore, 0–8 mg/mL LCP and FOS‐LCP and 5 mM CaCl_2_ were selected for subsequent experiments.

Although FOS‐LCP has good digestive stability, which can enhance calcium bioavailability, dietary inhibitors such as PA and OA readily combine with calcium ions, thereby reducing intestinal absorption (Wu et al. 2024). Moreover, divalent metal ions may compete for transport via the divalent metal transporter‐1 (DMT1), exerting inhibitory effects (Lin et al. 2020). Therefore, the protective role of FOS‐LCP should be evaluated under these conditions. In the absence of dietary inhibitors, both LCP and FOS‐LCP markably increased intracellular calcium compared with CaCl_2_ alone (*p* < 0.05; Figure 3B), enhancing absorption by 56.35% and 92.57%, respectively. Notably, FOS‐LCP showed greater efficacy (*p* < 0.05), suggesting that its absorption–promoting effect may involve additional pathways distinct from those of LCP (Wu, Wang, et al. 2023). When Zn^2+^ was added, calcium uptake declined markedly, but the presence of LCP or FOS‐LCP significantly alleviated this inhibition (*p* < 0.05). Interestingly, Fe^2+^ did not interfere with cellular calcium absorption (*p* > 0.05), implying that LCP‐ and FOS‐LCP‐mediated absorption may occur via pathways independent of DMT1 (Lin et al. 2020). As expected, when OA, PA, and PPA were added, the intracellular calcium concentration significantly decreased by 52.35%, 59.67%, and 66.64% (*p* < 0.05), respectively, confirming their inhibitory effects. However, supplementation with LCP or FOS‐LCP effectively prevented calcium precipitation and significantly improved cellular calcium uptake under these inhibitory conditions. Collectively, these findings indicate that the FOS‐LCP copolymer could overcome the adverse effects of dietary inhibitors, enhance calcium solubility, and promote calcium bioavailability.

#### 3.3.2 Calcium Transport Efficiency in the Intestine

Calcium transport across the intestinal epithelium involves two main pathways: paracellular and transcellular transport (Wu, Wang, et al. 2023). To further clarify how LCP and FOS‐LCP promote calcium absorption, an everted gut sac model was used (Figure 3C). When treated with 4 and 8 mg/mL LCP, the transported calcium was 1.84‐ and 1.85‐fold higher than that in the control, respectively (*p* < 0.05), with no significant difference between the two concentrations (*p* > 0.05; Figure 3D). This plateau effect suggests that LCP‐mediated calcium absorption reached saturation, consistent with the predominance of the transcellular transport pathway (Lin et al. 2020). Similar findings have been previously reported, showing that calcium‐chelating peptides primarily facilitate calcium transport through the transcellular pathway in an everted gut sac model (Sun, Wang, et al. 2020). In contrast, FOS‐LCP enhanced calcium transport in a concentration–dependent manner (*p* < 0.05). In the presence of 8 mg/mL FOS‐LCP, the transported calcium level was 2.42–fold higher than that in the control (*p* < 0.05). These findings indicate that FOS‐LCP facilitates calcium absorption primarily through the paracellular pathway (Wu, Wang, et al. 2023). Notably, the transported calcium content in the FOS‐LCP group was significantly higher than that in the LCP group (*p* < 0.05), implying that conjugation with FOS, an established prebiotic known to enhance calcium absorption, is a promising strategy for improving peptide–mediated calcium delivery.

### 3.4 FOS‐LCP Attenuated Adipogenesis While Enhancing Osteogenesis

**FIGURE 4:**
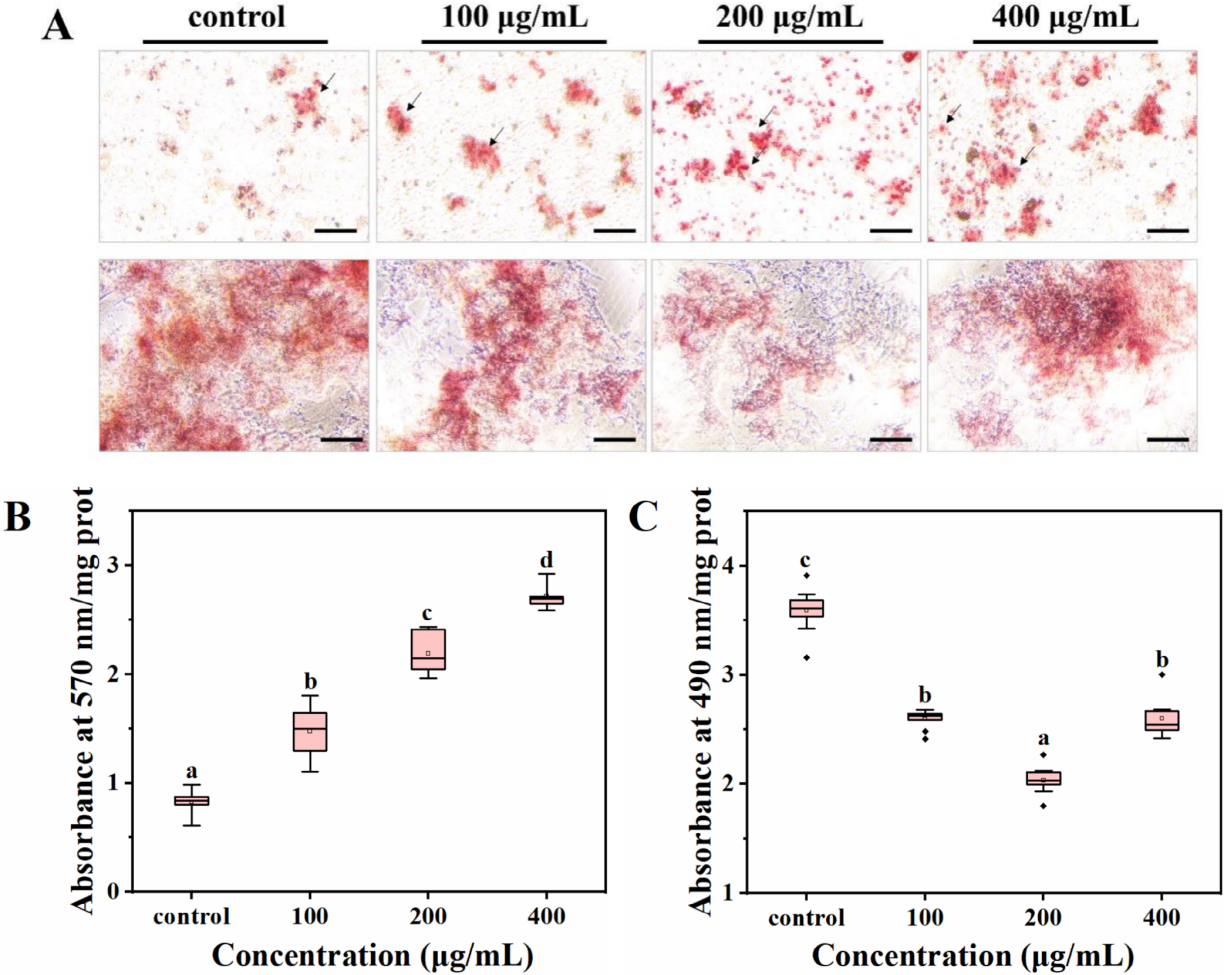
Effects of calcium delivery system on osteogenic‐adipogenic differentiation of bone marrow mesenchymal stem cells (BMSCs). (A) Representative staining images after 21 days of differentiation with different concentrations of FOS‐LCP (0–400 μg/mL) mixed with 5 mM CaCl_2_. Top panel: Alizarin red S staining; bottom panel: oil red O staining (control: 5 mM CaCl_2_ only); (B) quantification of mineralized calcium nodules; (C) quantitative oil red O staining analysis. Scale bar: 100 μm. All differentiation and quantification experiments were performed with three independent biological replicates (*n* = 3), each with technical triplicates. Different lowercase letters indicate significant differences among concentrations (*p* < 0.05).

BMSCs are the progenitors of osteoblasts and adipocytes in the bone marrow (Zhang et al. 2021). An increased number of adipocytes is often accompanied by a reduced number of osteoblasts, leading to impaired osteogenesis (Seok et al. 2020). To evaluate the regulatory role of the calcium delivery system in osteogenic and adipogenic differentiation at the cellular level, BMSCs were isolated from calcium‐deficient mice and differentiated in vitro. As shown by alizarin red S staining, the control group exhibited a limited mineralized matrix, reflecting a weak osteogenic function (*p <* 0.05; Figure 4A, top panel; Figure 4B). After FOS‐LCP treatment, the number of calcified nodules increased in a concentration‐dependent manner (*p <* 0.05), indicating progressive enhancement of osteogenic activity. In contrast, oil red O staining revealed strong adipogenic capacity in the untreated control group (Figure 4A, bottom panel; Figure 4C). After treatment with FOS‐LCP, adipocyte differentiation was significantly inhibited (*p <* 0.05). Comparable results were obtained for the MC3T3‐E1 cells and 3T3‐L1 preadipocytes (Figure S2). Collectively, these results suggest that the FOS‐LCP calcium delivery system can steer the osteogenic‐adipogenic lineage commitment of BMSCs under calcium‐deficient conditions.

### 3.5 Effect on BMSC Proliferation and Osteogenic Differentiation

**FIGURE 5:**
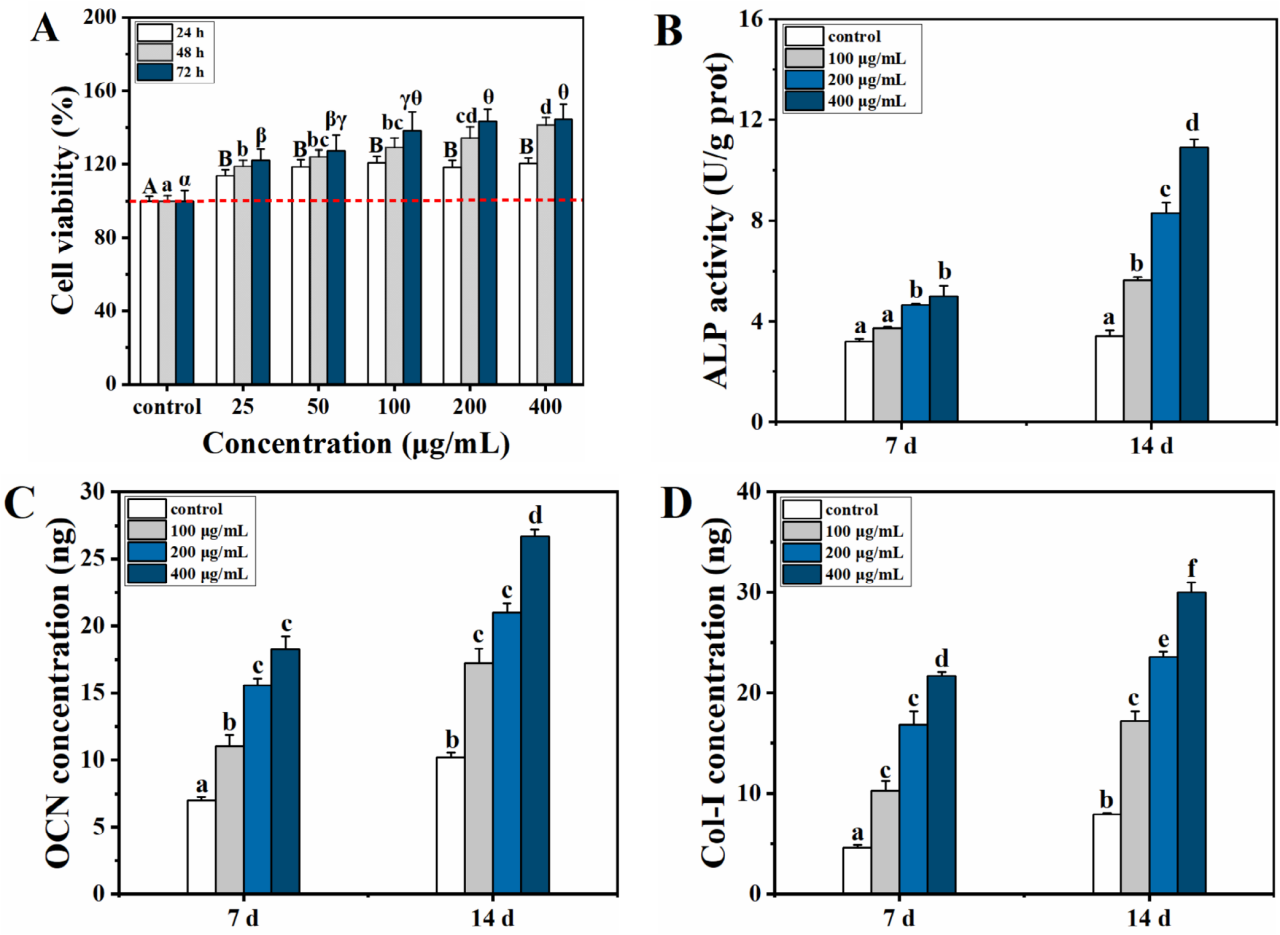
Effects of FOS‐LCP on proliferation and osteogenic differentiation of BMSCs (control: 5 mM CaCl_2_ only). (A) Cell viability of BMSCs treated with different concentrations of FOS‐LCP (25–400 μg/mL) mixed with 5 mM CaCl_2_ for 24, 48, and 72 h; (B) alkaline phosphatase (ALP) activity after 7 and 14 days of differentiation; (C) osteocalcin (OCN) secretion levels after 7 and 14 days of differentiation; (D) type I collagen (Col‐I) secretion levels after 7 and 14 days of differentiation. All differentiation and quantification experiments were performed with three independent biological replicates (*n* = 3), each with technical triplicates. Different lowercase letters indicate significant differences among concentrations or time points within the same treatment group (*p* < 0.05).

To clarify the osteogenic regulatory effects of the calcium delivery system, we explored its role in BMSC osteogenesis. Osteoblast proliferation and differentiation are prerequisites for new bone formation and critical for maintaining bone metabolism and homeostasis (Wu et al. 2022). After treatment with FOS‐LCP, BMSC viability increased markedly (Figure 5A); particularly, the cell viability after treatment with 400 μg/mL FOS‐LCP for 72 h was 144.73% of that after the control treatment. These findings indicate that this calcium delivery system can effectively promote BMSC proliferation and support bone formation (Wu et al. 2022). During differentiation, osteoblasts produce extracellular matrix proteins such as Col‐I, ALP, and OCN, which facilitate matrix mineralization and influence adipogenesis (Seok et al. 2020). Treatment with FOS‐LCP for 7 or 14 days significantly increased ALP activity as well as OCN and Col‐I production in a concentration–dependent manner (*p <* 0.05; Figure 5B–D). ALP is indicative of early osteoblast differentiation, whereas OCN and Col‐I are markers of osteoblast maturation and late differentiation, respectively. Col‐I secretion inhibits adipocyte differentiation (Liu et al. 2022). Taken together, these results suggest that FOS‐LCP effectively facilitates osteoblast differentiation at both early and mature stages, while simultaneously exerting a potential suppressive effect on adipocyte differentiation, thereby supporting bone formation.

### 3.6 Expression of Wnt/β‐Catenin Pathway‐Related Factors

Enhanced calcium absorption is not only fundamental for bone matrix mineralization, but also provides a signaling basis for activating the Wnt/β‐catenin pathway (Vannucci et al. 2018). Elevated intracellular Ca^2+^ levels can facilitate the glycosylation of low‐density lipoprotein receptor–related protein 5/6 (LRP5/6), thereby stabilizing its interaction with Wnt3a ligands (Wu et al. 2022). Concurrently, increased intracellular Ca^2+^ promotes the phosphorylation–dependent inactivation of glycogen synthase kinase‐3 beta (GSK‐3β) via Ca^2+^‐sensitive kinases such as Ca^2+^/calmodulin‐dependent protein kinase II (CaMKII) and protein kinase C (PKC) (Hu et al. 2024; Ren et al. 2021). These events collectively prevent β‐catenin degradation, promote its nuclear translocation, and ultimately activate osteogenic genes, including runt‐related transcription factor 2 (Runx2) and Col‐I (Hu et al. 2024; Wu et al. 2022). Simultaneously, Wnt/β‐catenin pathway activation suppresses adipogenic lineage commitment by downregulating key transcription factors such as peroxisome proliferator‐activated receptor gamma (PPARγ) and CCAAT/enhancer‐binding protein alpha (C/EBPα) and inducing effectors such as matrix metalloproteinase‐7 (MMP7), which further attenuates lipid accumulation (de Winter and Nusse 2021). Notably, calcium deficiency has been linked to impaired Wnt/β‐catenin activity and consequent reductions in bone formation (Sheng et al. 2017). Thus, by improving calcium bioavailability, the FOS‐LCP copolymer provides both substrate and signaling conditions to promote osteogenesis while suppressing adipogenesis in calcium‐deficient conditions.

**FIGURE 6:**
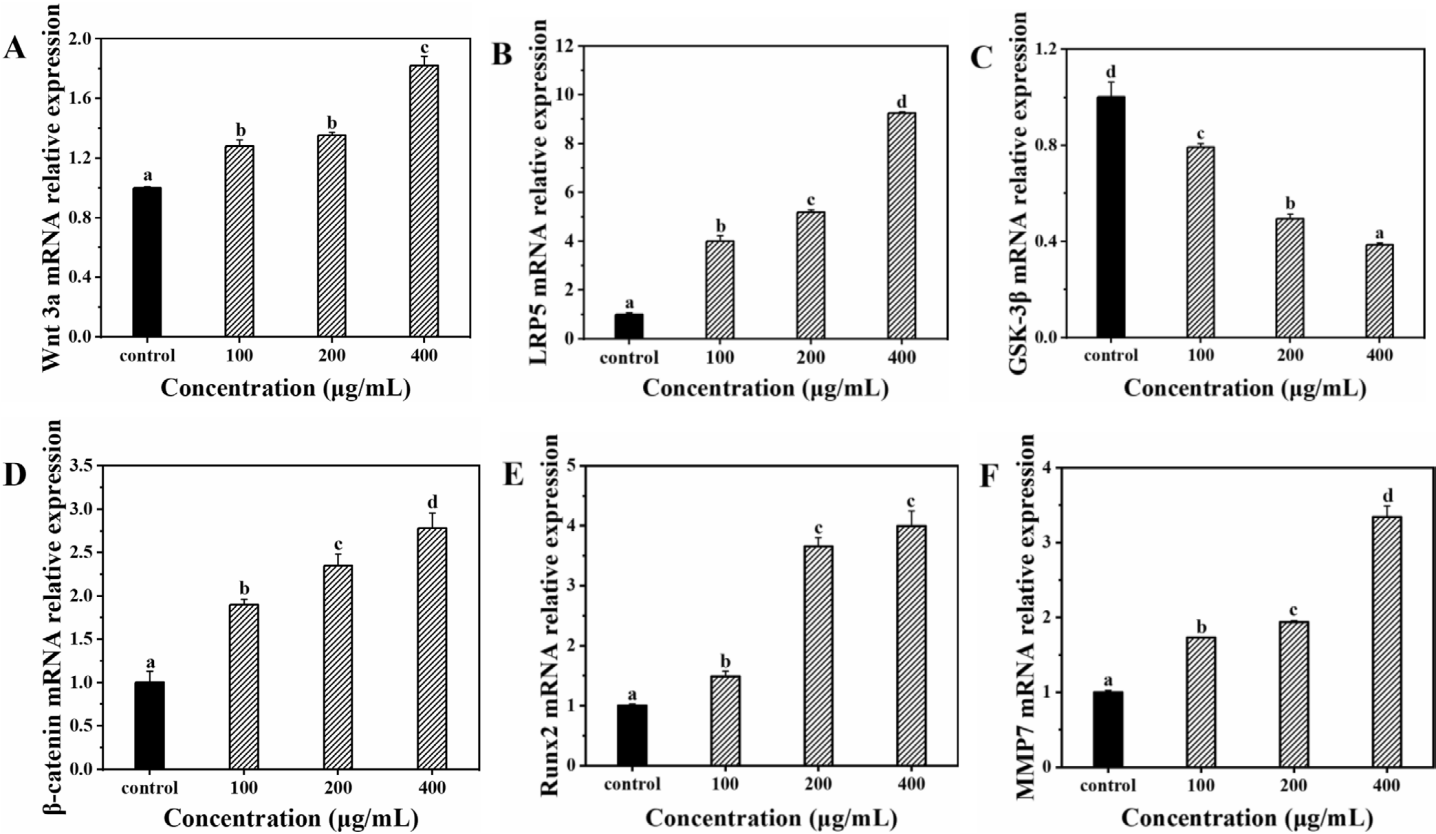
The mRNA expression of Wnt/β‐catenin signaling pathway‐related factors after osteogenic differentiation of BMSCs for 7 d in the presence of 0–400 μg/mL FOS‐LCP mixed with 5 mM CaCl_2_ (control: 5 mM CaCl_2_ only). All differentiation and quantification experiments were performed with three independent biological replicates (*n* = 3), each with technical triplicates. Different lowercase letters indicate significant differences among concentrations (*p* < 0.05).

To substantiate this mechanistic link, we examined the expression of canonical Wnt/β‐catenin signaling factors in BMSCs after FOS‐LCP treatment. The Wnt3a, LRP5, β‐catenin, Runx2, and MMP7 mRNA expression levels were significantly upregulated, whereas GSK‐3β expression was markedly reduced in a concentration–dependent manner (*p* < 0.05; Figure 6). Moreover, pretreatment with Dickkopf–1 (DKK1), a specific Wnt/β‐catenin inhibitor, diminished Col‐I secretion and shifted key pathway factors toward control levels (Figure S3), indicating that the canonical Wnt/β‐catenin pathway is required for the FOS‐LCP effect, although a direct causal dependence on Ca^2+^ influx remains to be demonstrated.

### 3.7 Effects on Intracellular Lipid Content

**FIGURE 7:**
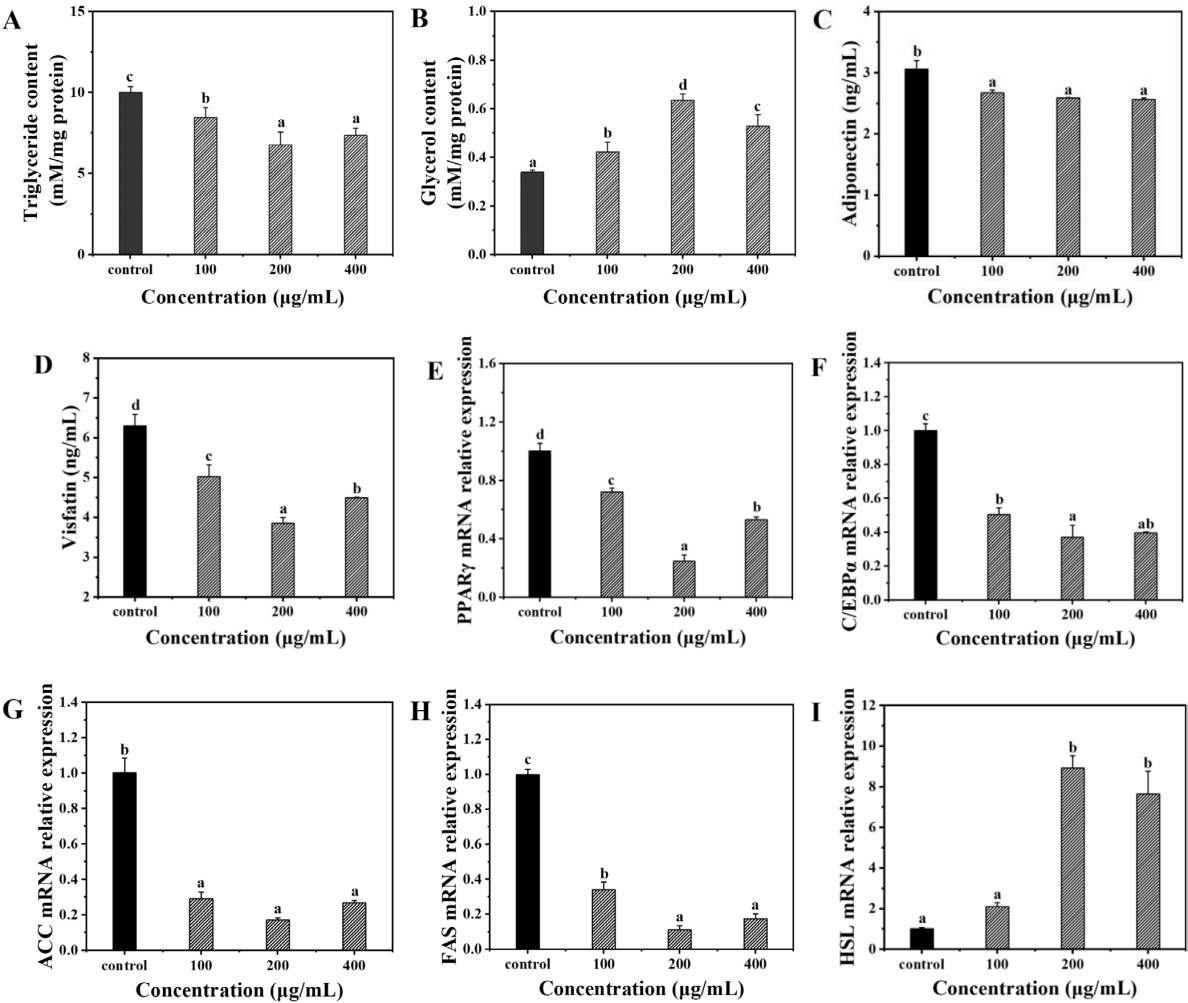
Effects of FOS‐LCP on adipogenesis of BMSCs after 10 days of differentiation in the presence of 0–400 μg/mL FOS‐LCP mixed with 5 mM CaCl_2_ (control: 5 mM CaCl_2_ only). (A) Intracellular triglyceride content; (B) glycerol content in the culture medium; (C) adiponectin secretion; (D) visfatin secretion; (E, F) relative mRNA expression of peroxisome proliferator–activated receptor gamma (PPARγ) and CCAAT/enhancer‐binding protein alpha (C/EBPα); (G, H) relative mRNA expression of acetyl‐CoA carboxylase (ACC) and fatty acid synthase (FAS); (I) relative mRNA expression of hormone‐sensitive lipase (HSL). All differentiation and quantification experiments were performed with three independent biological replicates (*n* = 3), each with technical triplicates. Different lowercase letters indicate significant differences among concentrations (*p* < 0.05).

Next, we sought to clarify the lipid‐lowering activity of FOS‐LCP in BMSCs to verify its role in the bidirectional regulation of osteogenesis and adipogenesis. The lipid content of adipocytes depends on the equilibrium between lipogenesis and lipolysis (Chalise et al. 2019). The intracellular TG content was used to analyze lipid accumulation, whereas lipolysis was determined by quantifying the glycerol content in the culture medium (Wu, Wang, et al. 2023). Compared with the control, FOS‐LCP (200 μg/mL) reduced the cellular TG content by 32.27% (*p* < 0.05; Figure 7A), consistent with the earlier oil red O staining results (Figure 4A,C). In parallel, glycerol content in the FOS‐LCP‐treated group reached 0.64 mM/mg protein, which was 1.88‐fold higher than that in the control group (*p* < 0.05; Figure 7B). These data clearly show that FOS‐LCP reduces lipid accumulation while accelerating glycerol release, thereby inhibiting adipogenesis.

Adipocytes also secrete bioactive adipokines such as adiponectin and visfatin, which act in autocrine and paracrine ways to promote adipogenesis and lipid accumulation (Zhang et al. 2021). FOS‐LCP treatment significantly decreased the secretion of both adiponectin and visfatin (*p* < 0.05), with 200 μg/mL FOS‐LCP producing the strongest inhibitory effect (Figure 7C,D). Taken together, the calcium delivery system is capable of inhibiting the differentiation of BMSCs into adipocytes under calcium‐deficient conditions.

### 3.8 Effects on Lipid Metabolism‐Related Genes in Adipocytes

PPARγ and C/EBPα are not isolated transcriptional regulators, but function in a synergistic molecular network to initiate and maintain preadipocyte differentiation (Chalise et al. 2019; Wang et al. 2016). PPARγ acts as a master switch that activates the transcription of downstream adipogenic genes such as aP2 and SREBP‐1c, whereas C/EBPα reinforces adipogenic commitment by binding to the PPARγ promoter and enhancing its expression, thereby forming a positive feedback loop that amplifies the differentiation program (Lee et al. 2019). FOS‐LCP treatment significantly downregulated the expression of both PPARγ and C/EBPα compared with the control (*p <* 0.05; Figure 7E,F). This dual suppression likely disrupts their reciprocal activation: reduced PPARγ weakens C/EBPα transcriptional activity, while diminished C/EBPα fails to further upregulate PPARγ, thereby breaking the adipogenic feedback loop (Seok et al. 2020). As reported in previous studies, this coordinated downregulation directly impaired adipogenesis initiation and subsequent lipid accumulation (Chalise et al. 2019). Moreover, PPARγ is a key regulator of adiponectin synthesis, so the decreased adipokine secretion observed after FOS‐LCP treatment may be a direct downstream effect of PPARγ inhibition (Abd Rami et al. 2023). Notably, this regulatory pattern is consistent with the FOS‐LCP–induced canonical Wnt/β‐catenin pathway activation, as Wnt/β‐catenin signaling can antagonize PPARγ transcription via TCF/LEF binding sites on the PPARγ promoter (de Winter and Nusse 2021). Taken together, the Wnt/β‐catenin activation and PPARγ/C/EBPα suppression support a model where FOS‐LCP attenuates BMSC adipogenic differentiation by disrupting the PPARγ/C/EBPα synergistic regulatory network.

Lipid accumulation and lipolysis in adipocytes are tightly controlled by a hierarchical molecular axis involving transcription factors and lipid‐metabolizing enzymes (Wu, Wang, et al. 2023). To clarify the downstream molecular interactions of PPARγ/C/EBPα suppression, we further analyzed the expression of key lipid metabolism–related genes (Chalise et al. 2019). The relative mRNA expression of lipogenic enzymes (including acetyl–CoA carboxylase [ACC], fatty acid synthase [FAS], adipocyte fatty acid binding protein [aP2], and sterol regulatory element‐binding protein‐1c [SREBP‐1c]) decreased significantly after FOS‐LCP treatment (*p* < 0.05; Figures 7G,H and S4A,B). Mechanistically, SREBP‐1c is a direct downstream target of PPARγ, and its downregulation should contribute to the reduced expression of ACC and FAS, which are rate‐limiting enzymes in de novo fatty acid synthesis under the transcriptional control of SREBP‐1c (Li et al. 2023; Vu et al. 2024). Meanwhile, aP2 is a well‐characterized PPARγ target involved in intracellular fatty acid transport, so its reduced expression further confirms impaired PPARγ‐mediated adipogenic signaling (Tang et al. 2023).

Similarly, FOS‐LCP treatment significantly upregulated the expression of key lipolytic enzymes (including hormone‐sensitive lipase [HSL], carnitine palmitoyltransferase 1 [CPT1], and adipose triglyceride lipase [ATGL]; *p <* 0.05; Figures 7I and S4C,D). ATGL initiates triglyceride hydrolysis by cleaving triacylglycerols into diacylglycerols, HSL catalyzes the subsequent breakdown of diacylglycerols to free fatty acids, and CPT1, the rate‐limiting enzyme for fatty acid β–oxidation, transports free fatty acids into the mitochondrial matrix (Li et al. 2022). Intracellular calcium activates the AMP‐activated protein kinase (AMPK) pathway, which, in turn, stimulates lipolysis and fatty acid oxidation by modulating HSL/ATGL activity and CPT1 expression (Li et al. 2022). Since FOS‐LCP serves as a calcium delivery system, calcium‐dependent AMPK activation may have contributed to the observed upregulation of HSL, ATGL, and CPT1 in our model, although this mechanism requires further verification. This molecular link explains the increased glycerol release into the culture medium (a hallmark of lipolysis) (Figure 7B) and suggests that FOS‐LCP modulates the lipogenesis‐lipolysis balance by suppressing the PPARγ/C/EBPα/SREBP‐1c axis, thereby reducing fatty acid synthesis and transport and enhancing triglyceride hydrolysis. Together, these molecular interactions attenuate intracellular lipid accumulation in BMSC‐derived adipocytes.

**FIGURE 8:**
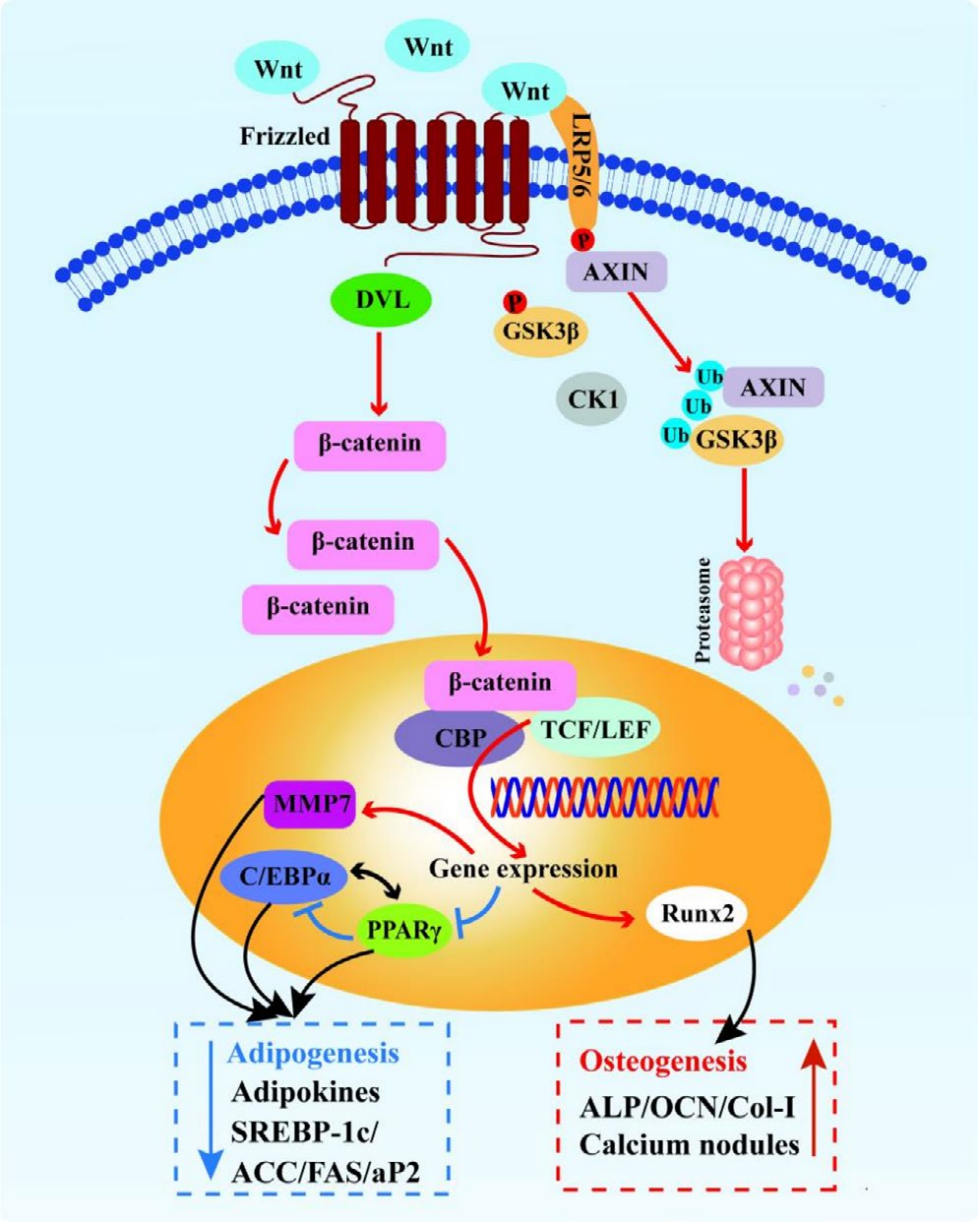
Proposed mechanism of bone‐lipid metabolic regulation by FOS‐LCP via the Wnt/β‐catenin pathway. In the presence of the FOS‐LCP copolymer, Wnt ligands activate the Frizzled receptor and the co‐receptor low‐density lipoprotein receptor‐related protein 5/6 (LRP5/6), leading to activation of disheveled (DVL) and inactivation of the β‐catenin destruction complex (axis inhibition protein [AXIN]/adenomatous polyposis coli [APC]/glycogen synthase kinase 3β [GSK‐3β]). Stabilized β‐catenin accumulates and translocates to the nucleus, where it cooperates with T‐cell factor (TCF)/lymphoid enhancer factor (LEF) and CREB‐binding protein (CBP) to drive target‐gene transcription. Osteogenesis: runt‐related transcription factor 2 (Runx2) is upregulated, enhancing early ALP activity and the initiation of extracellular matrix mineralization, promoting Col‐I synthesis to build the organic scaffold, and inducing OCN as a late marker—together driving BMSC commitment to the osteoblastic lineage. Anti‐adipogenesis: matrix metalloproteinase 7 (MMP7) is upregulated, which remodels the adipogenic niche; PPARγ and C/EBPα are repressed, attenuating downstream lipogenic gene expression (FAS, ACC, adipocyte fatty acid‐binding protein (aP2), sterol regulatory element‐binding protein 1c [SREBP‐1c]), while lipid–catabolic enzymes (HSL, adipose triglyceride lipase [ATGL], carnitine palmitoyltransferase 1 [CPT1]) are increased, reducing triglyceride content and lipid droplet accumulation. Overall, FOS‐LCP enhances bone formation while suppressing adipogenesis.

Collectively, our data support a framework where the FOS‐LCP calcium delivery system exerts dual regulatory effects (Figure 8). It promotes osteogenesis by stimulating osteogenic‐related factor secretion and activating the Wnt/β‐catenin signaling pathway, while concurrently suppressing adipogenesis by activating downstream targets of the Wnt/β‐catenin signaling pathway (such as MMP7), downregulating PPARγ and C/EBPα, modulating key lipid‐metabolic enzymes, and reducing adipokine secretion.

## 4 Conclusion

In this study, an FOS‐LCP copolymer was successfully prepared using a wet‐heating treatment for use as a novel calcium delivery system. The physicochemical characteristics and biological activities related to calcium absorption and bone‐lipid metabolism regulation were thoroughly evaluated. These results demonstrate that FOS‐LCP exhibited excellent calcium‐binding capacity, thermal stability, and resistance to enzymatic digestion, effectively mitigating the adverse effects of dietary inhibitors on calcium absorption and enhancing calcium bioavailability. Moreover, the FOS‐LCP copolymer significantly promoted osteogenesis and suppressed adipogenesis in BMSCs isolated from calcium‐deficient mice under in vitro calcium‐deficient conditions. These beneficial effects were associated with Wnt/β‐catenin signaling pathway activation and downstream osteogenic target upregulation. Concurrently, adipogenesis was attenuated by downregulating key adipogenic transcription factors (PPARγ and C/EBPα), reducing adipokine secretion, and modulating lipase expression. This study provides substantial theoretical support for the development of peptide‐polysaccharide calcium delivery systems as functional food ingredients to address calcium deficiency and bone‐lipid metabolism‐related disorders. However, long‐term in vivo intervention studies with oral FOS‐LCP supplementation and a comprehensive assessment of serum and bone density indicators are required to confirm its systemic efficacy at the whole‐animal level.

## Author Contributions

**Chunlei Liu:** conceptualization (equal), formal analysis (equal), investigation (equal), methodology (supporting), writing – original draft (supporting). **Xiaoping Wu:** conceptualization (lead), formal analysis (equal), funding acquisition (equal), investigation (equal), methodology (lead), writing – original draft (lead). **Yihang Guo:** formal analysis (equal), investigation (equal), methodology (equal). **Dan Li:** conceptualization (equal), methodology (equal), writing – review and editing (equal).

## Ethics Statement

All animal procedures were approved by the Animal Care and Use Committee of Ningde Normal University (approval number: NDNU‐LL‐202507).

## Conflicts of Interest

The authors declare no conflicts of interest.

## Supporting information


**Data S1:** fsn371415‐sup‐0001‐supinfo.docx.

## Data Availability

The data that support the findings of this study are available from the corresponding author upon reasonable request.
