# Supplementary material for: Fabrication of Fructooligosaccharide‐Larimichthys crocea Peptide Copolymer by Wet‐Heating for Enhanced Calcium Delivery and Bone‐Lipid Metabolism Regulation
Source: Food Sci Nutr. 2026 Jan 2;14(1):e71415. doi: 10.1002/fsn3.71415 (PMC12759107; doi:10.1002/fsn3.71415)
Supplement: Supplementary file 1 — Data S1: fsn371415‐sup‐0001‐supinfo.docx. [file FSN3-14-e71415-s001.docx]

**Supporting information**

**Table S1** Body weight of mice fed on a calcium-deficient diet

| **Order number** | **4th week body weight of mice/g** | **8th week body weight of mice/g** |
| --- | --- | --- |
| 1 | 30.37 | 34.76 |
| 2 | 30.51 | 30.37 |
| 3 | 33.84 | 36.68 |
| 4 | 27.90 | 32.87 |
| 5 | 37.42 | 31.17 |

**Table S2** PCR primer sequences

| **Analyte** | **Forward Primer(5’-3’)** | **Reverse Primer(5’-3’)** |
| --- | --- | --- |
| **18S** | ATGCGGCGGCGTTATTCC | CTGTCAATCCTGTCCGTGTC |
| **Wnt3a** | CTGACTGGAAGAGCGGAGAG | GACGGCTGAGTAGGGAACAC |
| **β-catenin** | GATTTCAAGGTGGACGAGGA | CACTGTGCTTGGCAAGTTGT |
| **LRP5** | TGCCCTGAAACTCCAAAAGC | CTTCACGCCACACAAGTAGG |
| **GSK-3β** | TGAGGACCATCTTTCTGCTCA | TGGTCTGATAGCTCGTCACA |
| **MMP7** | GAGTGAGCTACAGTGGGAACA | CTATGACGCGGGAGTTTAACAT |
| **Runx2** | AGATGGGACTGTGGTTACCG | TAGCTCTGTGGTAAGTGGCC |
| **PPARγ** | GACATTCCATTCACAAGAGC | TTCAGAATAATAAGGTGGAGATGC |
| **C/EBPα** | CAAGAACAGCAACGAGTACCG | GTCACTGGTCAACTCCAGCAC |
| **ACC** | CAGCATCTCTAACTTCCTTCAC | CGAGCCATTCATTATCACTACG |
| **FAS** | ATTCGGTGTATCCTGCTGTC | GCTTGTCCTGCTCTAACTGG |
| **HSL** | ACTGAGATTGAGGTGCTGTC | AGGTGAGATGGTAACTGTGAG |
| **aP2** | CCACAATCATCTGCTTCC | CTCATGCCCTTTCATAAACT |
| **SREBP-1C** | AGATGGGACTGTGGTTACCG | CCCACTGACCTTCTTCCC |
| **CPT1** | AGACAGACACCATCCAACAC | AGCCAGACCTTGAAGTAACG |
| **ATGL** | ATGGTGGCATTTCAGACAACC | CGGACAGATGTCACTCTCGC |


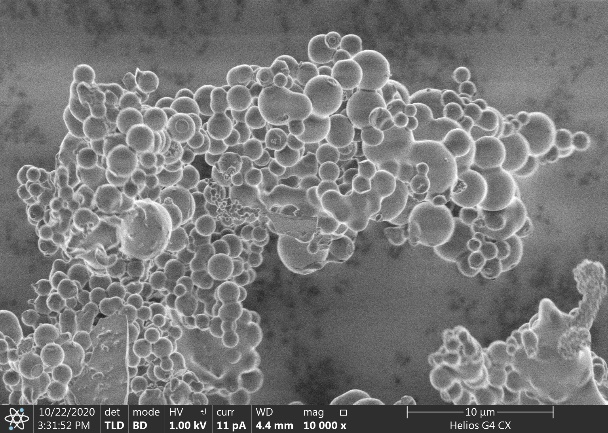

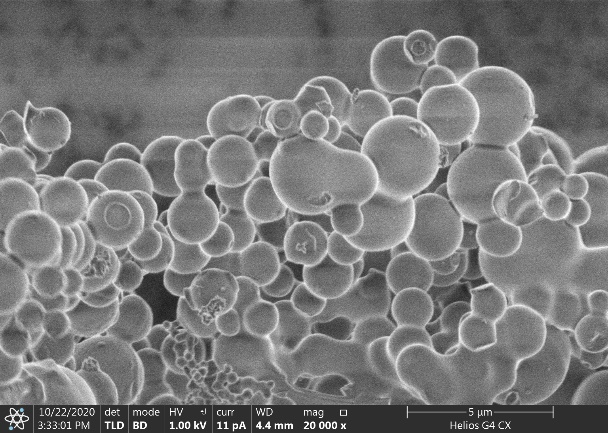


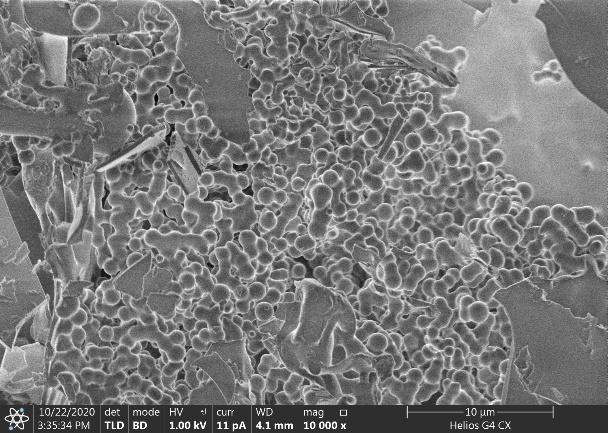

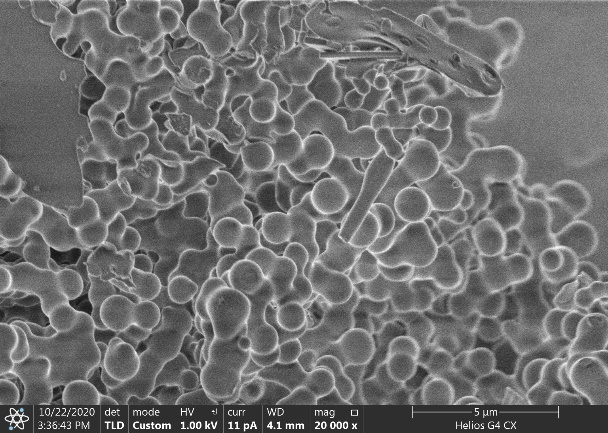


**Fig. S1.** Original SEM images of lyophilized LCP and FOS–LCP powders with instrument-generated scale bars. Lyophilized powders of *Larimichthys crocea* peptides (LCP) and fructooligosaccharide–Larimichthys crocea peptide (FOS–LCP) copolymer were examined using scanning electron microscopy (SEM) (Helios G4 CX, FEI, Brno, Czech Republic) under the same conditions as in the main text (accelerating voltage: 5 kV; working distance: 8–10 mm). Images were acquired at magnifications of 10000× (left column) and 20000× (right column), with the top panel showing LCP and the bottom panel showing FOS–LCP. The scale bars in these images are the original ones automatically generated by the SEM instrument (unmodified), corresponding to Fig. 2C in the main text (where scale bars were redrawn for clarity).


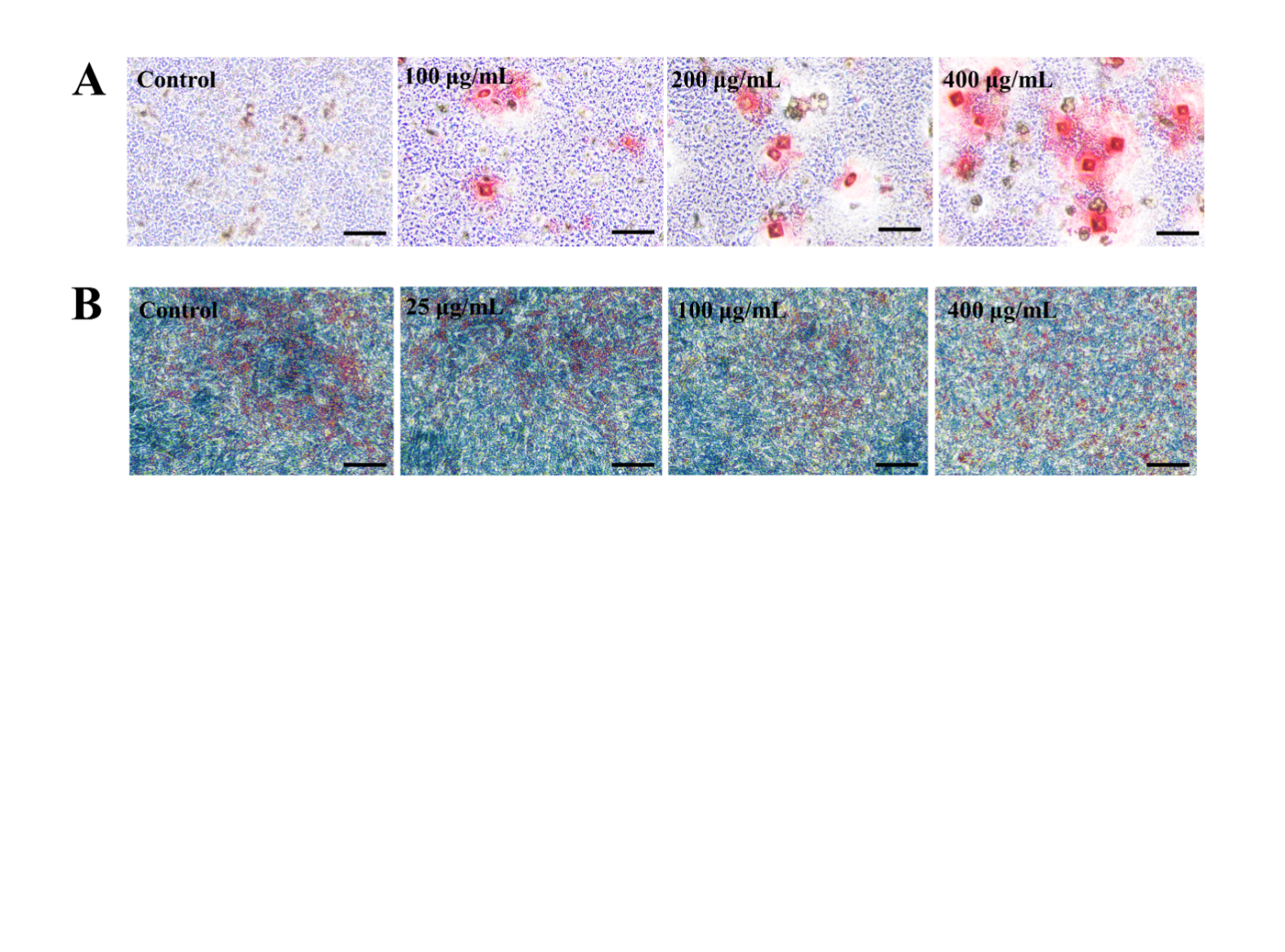



 **

**

**Fig. S2** Effects of calcium delivery system on osteogenic differentiation of MC3T3-E1 cells and adipogenic differentiation of 3T3-L1 cells. Control: 5 mM CaCl_2_ only. (A) Alizarin red S staining images of MC3T3-E1 cells differentiated for 21 days in inducers containing 0-400 μg/mL FOS–LCP mixed with 5 mM CaCl_2_; (B) Oil red O staining images of 3T3-L1 cells differentiated for 10 days in inducers containing 0-400 μg/mL FOS–LCP mixed with 5 mM CaCl_2_; (C) Quantification of mineralized calcium nodules: cells were destained with 10% (w/v) hexadecylpyridinium chloride in the dark for 1 h, and absorbance of eluted dye was measured at 570 nm; (D) Quantification of oil red O: stained cells were incubated with isopropanol at room temperature for 10 min with gentle agitation, and absorbance was measured at 490 nm. Scale bar: 100 μm. All experiments were performed with three independent biological replicates (*n*=3), each with technical triplicates. Different lowercase letters indicate significant differences among concentrations (*p*<0.05).

**



**

**



**

**



**

**Fig. S****3** Effects of the Wnt/β-catenin signaling pathway inhibitor Dickkopf-related protein 1 (DKK1) on the differentiation of bone marrow mesenchymal stem cells (BMSCs). BMSCs were preincubated with different concentrations of DKK1 (0-400 ng/mL) for 30 min, followed by 7-day induction of differentiation with an inducer containing 400 μg/mL FOS-LCP mixed with 5 mM CaCl₂. Control: 5 mM CaCl₂ only. (A) Type I collagen (Col-I) secretion; (B) Relative mRNA expression of Wnt family member 3a (Wnt3a); (C) low-density lipoprotein receptor-related protein 5 (LRP5); (D) catenin beta 1 (β-catenin); (E) Runt-related transcription factor 2 (Runx2); (F) matrix metalloproteinase 7 (MMP7). All experiments were performed with three independent biological replicates (*n*=3), each with technical triplicates. Different lowercase letters indicate significant differences among concentrations (*p*<0.05).

**



**

**



**

**Fig. S4** Effect of 10-day induction of BMSCs differentiation with an inducer containing 0-400 μg/mL FOS–LCP mixed with 5 mM CaCl_2_ on their lipid-metabolizing enzymes. Control: 5 mM CaCl₂ only. (A) Relative mRNA expression of adipocyte fatty acid-binding protein (aP2); (B) sterol regulatory element-binding protein 1c (SREBP-1c); (C) carnitine palmitoyltransferase 1 (CPT1); (D) adipose triglyceride lipase (ATGL). All experiments were performed with three independent biological replicates (*n*=3), each with technical triplicates. Different lowercase letters indicate significant differences among concentrations (*p*<0.05).
